# Carer perspectives on overweight, obesity and dental caries in early childhood: findings from a systematic qualitative review

**DOI:** 10.3389/froh.2025.1524715

**Published:** 2025-06-18

**Authors:** Heilok Cheng, Rebecca Chen, Bradley Christian, Jessica Appleton, Amit Arora, Elizabeth Denney-Wilson

**Affiliations:** ^1^ Faculty of Medicine and Health, Susan Wakil School of Nursing and Midwifery, The University of Sydney, Camperdown, NSW, Australia; ^2^ Sydney Institute of Women, Children and Their Families, Sydney Local Health District, NSW Health, Camperdown, NSW, Australia; ^3^ Translating Early Prevention of Obesity in Childhood (EPOCH-Translate) Centre of Research Excellence, The University of Sydney, Sydney, NSW, Australia; ^4^ Faculty of Medicine and Health, Westmead Applied Research Centre, The University of Sydney, Westmead, NSW, Australia; ^5^ Westmead Centre for Oral Health, Western Sydney Local Health District, NSW Health, Westmead, NSW, Australia; ^6^ Faculty of Medicine and Health, School of Dentistry, The University of Sydney, Camperdown, NSW, Australia; ^7^ Australian Centre for Integration of Oral Health, School of Nursing & Midwifery, Western Sydney University, Penrith, NSW, Australia; ^8^ Faculty of Health, School of Nursing and Midwifery, The University of Technology Sydney, Ultimo, NSW, Australia; ^9^ School of Health Sciences, Western Sydney University, Penrith, NSW, Australia; ^10^ Health Equity across Lifespan Research Laboratory, Campbelltown, NSW, Australia; ^11^ Translational Health Research Institute, Western Sydney University, Penrith, NSW, Australia; ^12^ Faculty of Medicine and Health, Sydney Medical School, The University of Sydney, Camperdown, NSW, Australia; ^13^ Oral Health Services, Sydney Local and Health District and Sydney Dental Hospital, Surry Hills, NSW, Australia

**Keywords:** dental caries, obesity, health knowledge, attitude and practice, child health, child nutrition sciences

## Abstract

**Introduction:**

Formula and bottle feeding behaviours can increase obesity and tooth decay (early childhood caries, ECC) in early childhood, through non-responsive feeding and prolonged exposure to sugar. Parents’ beliefs can be barriers to behaviour change for obesity and ECC prevention. Understanding these beliefs towards children's teeth and weight can address parents’ priorities and develop prevention messages. This qualitative systematic review (PROSPERO registration #CRD42022348783) aimed to identify parent or carer perspectives on obesity and ECC in children aged ≤6 years.

**Methods:**

Database searching of CINAHL, Medline and EMBASE, with hand searching, was undertaken. Included papers were qualitative research publications, focused on parent or carer beliefs and attitudes towards overweight, obesity or ECC in infants and children. Inductive thematic analysis was undertaken to generate themes, with a strengths-based approach focused on parents’ lived experience. Quality appraisal was undertaken with the CASP Qualitative Checklist. Descriptive characteristics of the study and participants, and qualitative findings, were extracted qualitatively in NVivo.

**Results:**

7,365 references were identified from database and hand searching, with 98 references included for analysis. Three research themes were generated: (1) parenting to support child wellness, including healthy teeth and weight; (2) parents’ response to unwellness, including identifying symptoms, causes and protective factors for unhealthy weight and teeth; (3) information and resources needed to support healthy weight and teeth. There was high or potential risk of bias in qualitative methodology when studies did not address researcher-participant relationships or rigorous data analysis processes.

**Discussion:**

Findings highlight the need for strength-based messages for children's teeth and weight, increased understanding of formula and bottle feeding as obesity and ECC risk factors, and holistic approaches to care by dental and primary care professionals.

**Systematic Review Registration:**

https://www.crd.york.ac.uk/PROSPERO/view/CRD42022348783, PROSPERO CRD42022348783.

## 1 Introduction

Overweight, obesity and dental caries (tooth decay) in early childhood are preventable conditions that share an overlapping aetiology through infant nutrition and feeding. Early childhood caries (ECC) are dental caries occurring in children aged under 6 years, with one or more primary teeth affected by tooth decay, loss or fillings (1). Overweight and obesity in children under 5 years of age is defined respectively as two and three standard deviations above the World Health Organization weight-for-height growth standard median (2). Consequences of these conditions may be extensive, such as impaired eating and nutrition from poor dentition impacting growth; and poor oral health and dental pain impacting speech development, sleep, behaviour, play, learning and later school attendance and performance (3). Increased body weight may be linked with delays in mental and motor development (4). Further, the establishment of increased body weight or unhealthy dietary patterns in infancy may increase obesity and obesogenic risk factors in childhood and adulthood (5–7), with similar increase in risk of chronic diseases such as type 2 diabetes and cardiovascular disease (8).

In addition to known dietary and behavioural risk factors of overweight, obesity and ECC (9, 10), formula and bottle feeding may contribute to overfeeding and exposure to free sugars (11). These behaviours may include feeding to soothe, instead of feeding responsively to hunger; adding fermentable carbohydrates to bottles; and ongoing use of bottles, instead of transitioning to cups (11). Health professionals report that parental beliefs can be a barrier to behavioural changes, such as the preference for infants with large body size; the value of feeding, particularly formula feeding, to encourage infant sleep; lack of concern to the health of primary teeth; and poor parental knowledge and self-efficacy to support tooth brushing and healthy eating (12–15). Whilst poor health literacy and values can overlap, some parents may value and place an emphasis of one health condition over another.

There is an opportunity to tailor health messaging towards values parents may place on one condition over another. That is, for a parent who values their child's oral health instead of weight concerns, targeted messaging focused on healthy teeth may also reduce risk factors related to their child being overweight. Understanding the beliefs and perceptions of parents and carers towards overweight, obesity or ECC will identify parental priorities in the care of their infants and children. In turn, this will inform co-design of a common risk factor intervention with consistent messaging to support best-practice formula and bottle feeding behaviours that prevent overweight, obesity and ECC.

This qualitative systematic review aimed to identify parent or carer (hereafter, referred to as “parents”) perspectives on overweight, obesity or dental caries in infants and children aged under 6 years.

## 2 Methods

The qualitative systematic review focused on the lived experiences of parents of infants and children aged under 6 years, and their perspectives on overweight, obesity or dental caries in early childhood. This review is reported following the Preferred Reporting Items for Systematic reviews and Meta-Analyses (PRISMA) 2020 statement (16), with checklist in Supplementary Table S1, and was registered on PROSPERO (#CRD42022348783). Thematic analysis (17) was used for theme generation and analysis. A review protocol is not available.

### 2.1 Information sources

Reference searches were undertaken in August 2021, with a search update in January 2023 and August 2024. The database search strategy was structured using the Population/Concept/Context framework (Table 1). Databases CINAHL, Medline (via OvidSP) and EMBASE (via OvidSP) were searched with combinations of key terms for: infant or child; dental caries or overweight, obesity or excess body weight; parent or carer; beliefs, perspectives, knowledge, attitudes or culture; and qualitative research (Supplementary Tables S2–S7). All references were downloaded to Endnote 21 (Clarivate, 2013, Philadelphia, USA) for screening.

**Table 1:** Study inclusion and exclusion criteria.

Population/Concept/Context criteria	Inclusion criteria	Exclusion criteria
Population: parent/carer of infant/child	• Parent or carer of infants or children aged ≤6 years	*Parent/carer* • Pregnant women* • Professionals that work with infants or children aged ≤6 years*, e.g. ○ Healthcare providers ○ Teachers ○ Early education staff *Infant/child* • Infants or children aged >6 years, or where infant/child age cannot be determined* • Infants or children with identified abnormal growth conditions, e.g. ○ Malnutrition ○ Failure to thrive ○ Stunting ○ Developmental disability ○ Premature birth
Concept	• Perspectives on dental health in early childhood at ≤6 years, specific to early childhood caries or • Perspectives on overweight, obesity or unhealthy weight gain in early childhood at ≤6 years, specific to body weight or size	• Non-qualitative perspective of infant/child weight, e.g., visual body size scale (160) • Infant or child health research not specific to overweight, obesity or dental caries, e.g. ○ Dental or oral health not specific to early childhood caries, or with minimal dental caries outcomes ○ Unhealthy weight, without focus on body weight or size ○ Other infant or child feeding • Other infant or child obesity research without weight outcomes or with minimal body weight or size outcomes
Context	• Infants or children diagnosed with overweight, obesity or dental caries or • Population at increased risk of overweight, obesity or dental caries in early childhood	• Population at increased risk of undernutrition, failure to thrive or stunting or • Population with underlying clinical condition, e.g., disability or • Population not at increased risk of overweight, obesity or dental caries in early childhood
Other	• Research with qualitative component, e.g., semi-structured interviews, focus group	• Research with brief qualitative component, e.g., open answer question in survey • Research with no qualitative component, e.g., structured interview-based survey
	• Journal article • Published in peer-reviewed journal • Primary research	• Thesis/dissertations • Published in predatory journal • Not primary research, e.g. ○ Reviews ○ Conference abstracts
	• English language	• Language other than English

* Studies were included if data from pregnant women, carers of infants/children aged >6 years or health professionals, could be separated from carers of infants/children aged ≤6 years.

Forward and backward citation (18) was undertaken using the reference lists of included studies and citations via Google Scholar. Reviews focused on infant or child overweight, obesity or oral health were checked for relevant articles.

The literature on parental perspectives of childhood overweight and obesity is extensive. Sydney Local Health District, the authors' local health district, is a culturally and socioeconomically diverse area of New South Wales, with nearly half the population speaking a language other than English, higher rates of obesity in cultural groups, areas of extreme socioeconomic advantage and disadvantage (19, 20), and where preference for large body size is a barrier to infant obesity prevention (12), particularly amongst cultural groups. To focus the literature searched, only studies with specific reference to body size were included for analysis.

Data extracted from articles included first author, year of publication, country of data collection, data collection method, study population, population characteristics, and qualitative findings.

### 2.2 Thematic analysis

Thematic analysis was undertaken following the six steps outlined by Braun and Clarke (17). Thematic analysis enabled inductive theme generation and could centre the participant perspective.

Full text documents and relevant supplementary files were uploaded to NVivo version 14 (Lumivero, 2023). HC undertook familiarisation of the research by reading the articles 2–3 times. Iterative initial codes were drafted, then organised into a coding framework that was informed by the Capability-Opportunity-Motivation system of behaviour change (21), which proposes that capability, opportunity and motivation underpin behaviour and behaviour change. Following coding, thematic generation was undertaken by HC to produce themes and subthemes, which were discussed with all authors; then reviewed and refined into three final themes addressing both obesity and ECC, using a strengths-based approach towards parents' lived experience.

### 2.3 Quality appraisal

Critical appraisal was undertaken using the CASP Qualitative Checklist (22) to assess qualitative research methodology. Critical appraisal was summarised as tabulated data and visualisation in RevMan 5.4 (The Cochrane Collaboration, 2020).

### 2.4 Inter-rater reliability

Author HC undertook all Methods. Inter-rater reliability was undertaken, with a second reviewer (CR, EDW or JA) to screen full-text studies included for analysis; code ∼10% of included studies, following the coding framework; and undertake the CASP Qualitative Checklist appraisal for ∼10% of included studies.

This ensured that two reviewers determined the final studies for inclusion; supported consistent coding accuracy; and assessed critical appraisal consistently. Inter-rater reliability of CASP appraisal was scored with percentage agreement (23).

### 2.5 Reflexivity

Reflexivity involves critical interrogation of how knowledge production is informed by the researcher's values (17). HC is a dietitian, with a background in childhood obesity research. During familiarisation with the literature, varying perspectives between healthcare providers and parents was evident, including frustrations with health recommendations not being undertaken or difficulties faced in parenting and child rearing not being recognised. HC maintained research memos and positioned the parent perspective during coding, theme generation and analysis. The findings are focused on the parent perspective, instead of biomedical models of physiology and pathophysiology, and parent-reported resource needs, instead of researcher-inferred needs. Examples of excerpts with reflexive coding are available in Supplementary Table S8.

## 3 Results

### 3.1 Study selection

**Figure 1:**
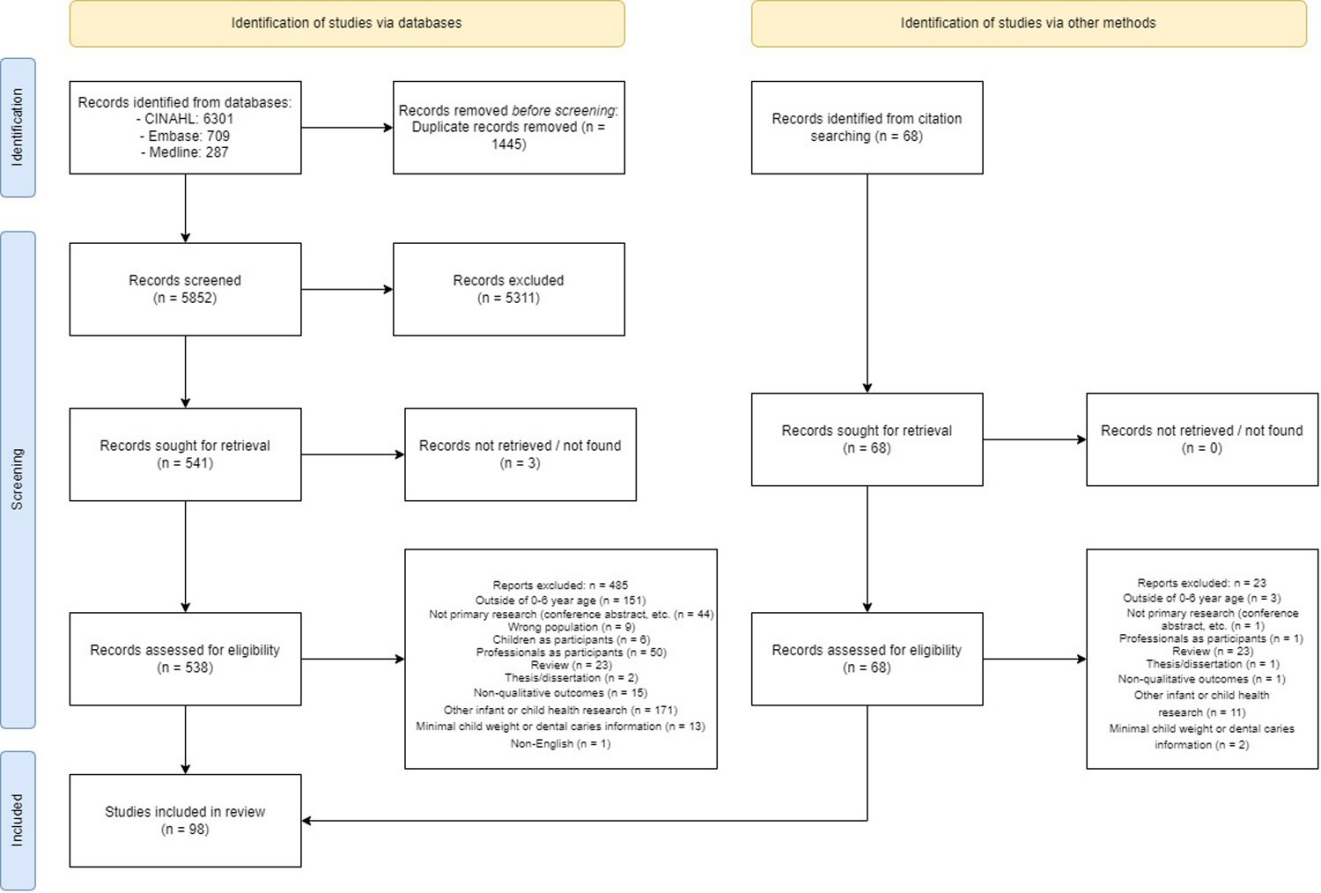
PRISMA flow diagram.

Results of the database and citation search are summarized in the PRISMA flow diagram (Figure 1). Database searching identified 7,297 references. Following duplicate removal, 5,852 references were screened for relevance based on title and abstract. 538 references were sought for full-text retrieval. Studies excluded during full-text screening included parents of children with unspecified age or outside of age range (24, 25); studies focused on feeding, nutrition and/or physical activity without context of weight, overweight/obesity or tooth decay (26, 27); weight-specific research with minimal focus on infant or child body size (28, 29); and dental research that was non-specific to feeding, nutrition and/or ECC (30, 31). Backward and forward citation searching identified 68 references. 98 articles from 91 studies were included.

### 3.2 Inter-rater reliability

Author HC independently undertook all Methods, including reference searching and screening; data extraction; study coding; and quality appraisal.

A second researcher, CR or JA, screened all full-text studies, against the inclusion and exclusion criteria. Disagreements were resolved by author EDW, with 98 articles included for analysis.

To ensure consistency in coding, eight articles were coded by EDW following the coding framework. Discussion between HC and EDW indicated high consistency in coding decisions.

Eleven studies were randomly selected for CASP assessment with a second reviewer, EDW or JA. There was high inter-rater reliability, with an overall 93.6% percentage agreement, and high agreement across all ten CASP checklist items (23) (Supplementary Table S9).

### 3.3 Included studies

Descriptive characteristics of the 98 articles are summarised in Supplementary Table S10. Most participants were mothers. Fifty-nine articles included infants and children with overweight, obesity or ECC, diagnosed by health professionals or self-reported by parents. Eighty-six articles involved populations at increased risk of overweight, obesity or ECC, relating to relative social disadvantage (low income; low education; refugees, migrants, immigrants or cultural minority populations; Indigenous populations), countries in economic transition, or parents with overweight/obesity. Most articles were from the USA (47), followed by European countries (12) and Australia (9). Studies from each continent were represented, mostly from North America (55), then Asia (13), Europe and Oceania (12 each), with fewer in South America (5) and Africa (1). All studies undertook qualitative research through focus groups (55) or interviews (56), barring one which used Photovoice.

### 3.4 Quality appraisal

CASP findings are summarised in Supplementary Table S11 and Figure S1. Overall, articles had good qualitative research quality: studies had clear research aims (*n* = 98); appropriate use of qualitative methodology (*n* = 98); results were valuable in relation to current practice or policy, transferability to other populations, or relevance to new areas of research (*n* = 96); and clearly stated findings for explicitness, credibility or relation to the research question (*n* = 82). Studies were at high or unclear risk of bias for not considering or explicitly reporting on the researcher-participant relationship (*n* = 64), the use of informed consent or sufficient explanation of the research process to participants (*n* = 44), and rigorous data analysis processes (*n* = 39).

### 3.5 Thematic analysis

**Figure 2:**
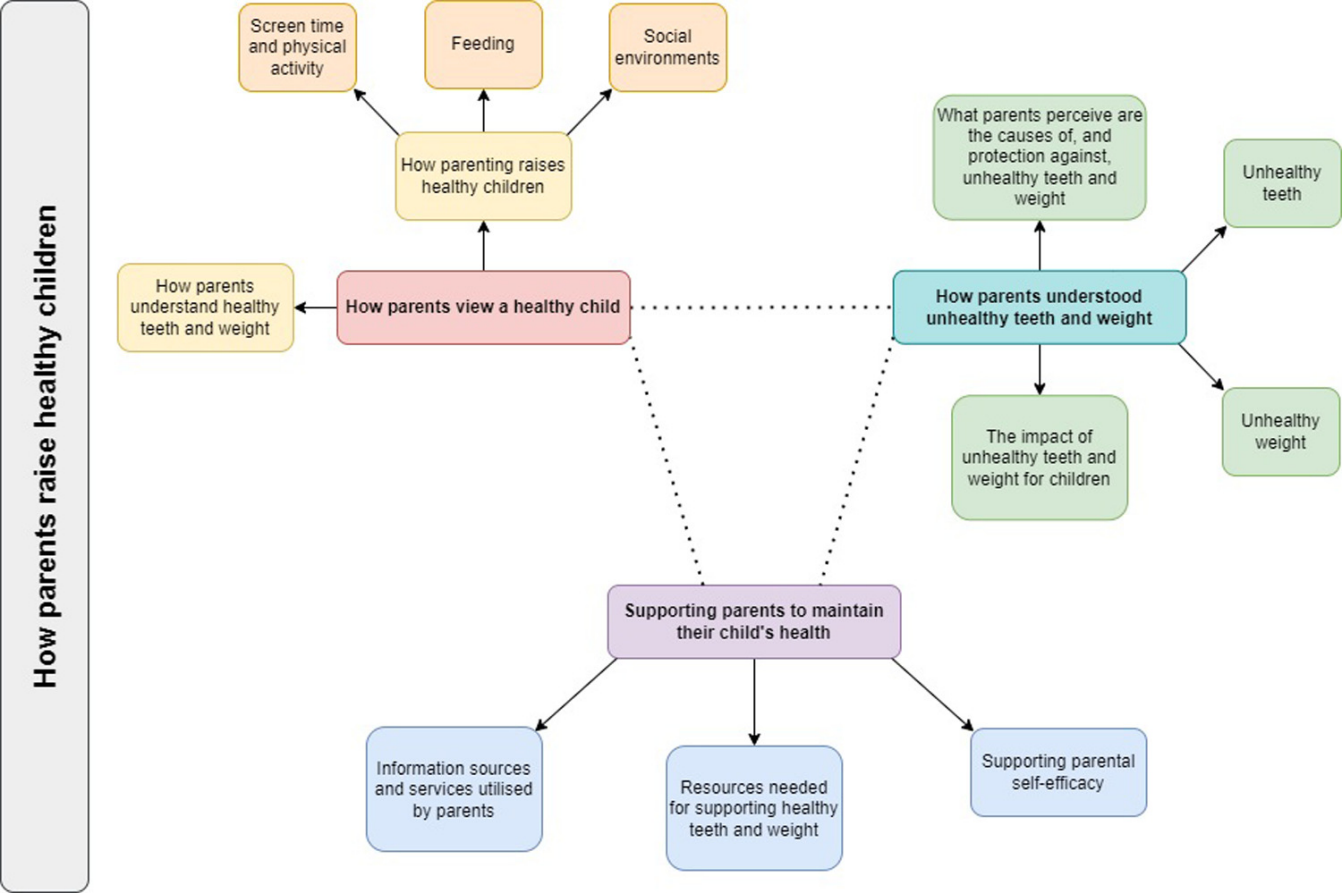
Thematic map.

Themes are conceptualised in Figure 2. Findings are grouped by how parents: value child *wellness*, including healthy teeth and weight, and the parenting practices supporting this (Theme 1); respond to *unwellness*, including identifying symptoms, causal factors and protective factors (Theme 2); and identify resources needed to support healthy teeth and weight (Theme 3)*.* Our findings demonstrate that although parents valued wellness, they tended to respond and seek resources only when children were unwell. This is consistent with existing literature around parental responses to health promotion when children are asymptomatic. Representative quotes are reported in Supplementary Table S12.

### 3.6 Theme 1: how parents view a healthy child

Good health for children was determined by observable physical, cognitive and emotional development, including happiness; self-esteem; good appetite, activity and play; life opportunities; growth and attaining developmental milestones; absence of illness and disease; learning ability; behaviour and temperament (32–43).

#### 3.6.1 How parents understand healthy teeth and weight

Good oral health was defined by the absence of disease: aesthetically pleasing teeth were white, straight, evenly spaced, without pain, holes, decay or discoloration; pink gums and attractive smiles (13, 41, 44–50). However, parents’ views on the health of primary and permanent teeth differed. Parents valued primary teeth if they believed it would impact the health of permanent teeth (47, 48, 51–53). Conversely, only permanent teeth were valued when these were viewed as having more long-term importance than primary teeth (49, 54–56).

Healthy or desirable body weight was determined by size or shape, appetite and physical activity. Many parents considered *largeness* or *heaviness* to demonstrate adequate nutrition and growth, energy reserves, physical strength, and contentment (35, 37, 39, 57–65). Parents considered good appetite and dietary intake, lack of fussiness around food, and physical activity or play without signs of overexertion or developmental delay, as signs of children having healthy weight, receiving adequate nutrition, and being sufficiently active (33, 35, 38, 39, 42, 43, 57–60, 62, 65–72). Largeness or heaviness, which demonstrated growth and nutrition, were distinct from overweight or obesity as disease conditions. This was conceptualised as “solid”, “thick”, or “fresh”, “baby fat”, “plumpness”, “chubbiness”, “muscled” or “chunkiness”, and could be informed by cultural beliefs and norms (32, 35, 38, 42, 43, 60, 62–66, 73, 74). Few studies found positive beliefs towards smaller body weight, and could be mitigated by health provider advice (67, 75) or beliefs about breastfeeding benefits (76).

Few parents viewed the use of growth charts as a clinical method of identifying healthy body weight positively (36, 40, 70, 77). However, parents also viewed high growth chart trajectories or percentiles positively when this was reported or viewed as healthy, “normal”, or showing increasing body weight (65, 78, 79).

#### 3.6.2 How parenting raises healthy children

Parents valued their ability to meet their children's wants and needs (43), and provide a safe family and household environment (33, 80, 81). These beliefs informed perspectives regarding feeding, activity and play, and screen time.

##### 3.6.2.1 Screen time and physical activity

Parents felt screen use could interfere with eating, appetite regulation and parent-infant bonding (58, 61, 71, 74, 82, 83). Screen use was associated with children being “lazy” or “inactive”, independent of health outcomes (33, 35, 60). However, screen time was believed to support learning, digital skill development and exposure to healthy foods (58, 84), and enabled children to behave indoors (61, 82, 85).

Parents felt physical activity was intrinsic in growth and development, with infants being naturally inclined to be active when they reached developmental milestones, such as crawling and walking (58, 86). Therefore, parents did not need to encourage play and activity. Similarly, for older children, parents only felt responsible for providing transport or safe play options (34, 87, 88). Parents felt they only needed to support physical activity if their child had overweight or obesity (69, 89).

##### 3.6.2.2 Feeding

Values towards healthy eating by children and feeding decisions from parents overlapped. While healthy eating supported physical and mental growth and contributed to a good appearance (37, 54, 58, 77, 90), this could include *forced feeding or pressure feeding practices* to ensure children were fed, regardless of hunger or appetite (36, 71, 74, 75, 77). Likewise, while feeding also demonstrated parental affection and the ability to meet children's physical and emotional needs (42, 62, 83, 91), this also meant offering cariogenic and/or obesogenic food being important to provide enjoyable eating experiences (74, 81) and to diversify a child's diet (53, 92). Denying this would deprive children of opportunities to enjoy food and learn self-regulation (54, 93). Early introduction of solid food to infants before six months broadened the taste palate to prevent fussy eating (70, 74).

##### 3.6.2.3 Social environments

Parenting approaches and the family environment supported oral hygiene and healthy eating, through supervising and/or modelling routine tooth brushing and food choices, making food purchasing decisions, ensuring healthy foods were available, limiting availability of unhealthy foods in the household, and avoiding pressuring children to eat foods they did not enjoy (33, 35, 37, 38, 41, 42, 45, 46, 49, 50, 52, 53, 61, 65, 67, 75, 77, 83, 84, 94–107).

Most parents considered their child's oral hygiene and dietary behaviours to be their responsibility. Broader social support were equally important, such as from other parents, older siblings and family members (84, 98–101, 103, 104, 107), with schools and healthcare providers also being responsible for teaching tooth brushing or providing healthy food environments (50, 67, 84, 87, 88, 96, 103, 107–109).

### 3.7 Theme 2: how parents understood unhealthy teeth and weight

Poor health and illness were defined by symptoms and health consequences that interfere with wellbeing, while early indicators of disease—such as white spots in ECC, or growth charts showing risk of overweight or obesity—could be overlooked or dismissed.

#### 3.7.1 Unhealthy teeth

Parents identified poor oral health through symptomatic disease and unattractive teeth. Few parents used medical terms, such as “tooth decay” or “cavities”—instead, this was reported as visible decay, including holes, “rot” and “black spots”; fractured or cracked teeth; tooth discoloration; tooth loss and extraction; pain, distress, poor behaviour and mood; bad breath; inability to eat and sleep; visible fillings; and swollen gums (36, 41, 44, 45, 48–51, 55, 100, 110–114).

#### 3.7.2 Unhealthy weight

Parents believed that smallness or thinness in children indicated undernutrition; poor growth and development; or illnesses, like anaemia and tapeworms. These beliefs could be informed by health provider assessment when children did not follow a standardised growth trajectory (32, 78) or in populations with poverty or socioeconomic disadvantage (33, 77, 96). Thinness was also associated with parental neglect or inability to meet nutritional needs (40, 66, 77, 96).

Parents considered infants or children to be at an unhealthy weight when their appearance or behaviour appeared unattractive; when they appeared “larger” than their peers; or when they required larger clothing sizes (35, 40, 59, 60, 62, 65, 66, 84, 89, 115, 116). While parents may acknowledge that their child had a clinical diagnosis of overweight or obesity, few parents felt this meant their child's weight was *unhealthy* unless physical and emotional consequences were experienced, which is discussed separately in theme 2.4.

Notably, there were opposing views on the suitability of standardised growth charts to identify healthy weight and growth. Few parents felt that growth charts were suitable for identifying overweight or obesity (35, 60, 67, 84, 115). Parents demonstrated uncertainty towards the purpose of growth charts (63, 70, 79). When growth charts identified a clinical diagnosis of overweight/obesity that was incongruent with parental expectations, parents viewed growth charts negatively or being incompatible with a child's unique growth trajectory (32, 35, 42, 79, 89).

#### 3.7.3 What parents perceive are the causes of, and protection against, unhealthy teeth and weight

Parents generally were able to identify certain causal and protective factors in ECC, overweight, obesity or weight gain. These findings are focused on the parental perspective, instead of biomedical models of pathophysiology.

For ECC, causal factors included insufficient tooth brushing, dietary sugar intake (particularly sweets, fruit juice and milk), and bottle feeding (13, 36, 44–53, 55, 76, 82, 85, 91, 95, 97, 99, 101, 103, 105, 107, 108, 110, 113, 117–119). Protective factors included regular tooth brushing or gum cleaning, limiting dietary sugar intake, limiting bottle use, and routine dental check-ups (41, 44, 46, 47, 49, 50, 52, 53, 98, 100, 103, 104, 107). Few parents identified night-time breastfeeding, cariogenic bacteria transmission, or carbohydrous foods, as causes of ECC (50, 53, 91, 95, 101, 105, 110); or the role of fluoride in ECC prevention (46, 47, 100, 104, 120).

For weight gain, causal factors included dietary intake of high fat and/or high sugar foods, overeating, early introduction of solid foods for infants, prolonged bottle or formula feeding, and physical inactivity or sedentary behaviour (35, 37, 60, 62, 65–67, 75, 77, 78, 82, 85, 86, 89, 90). Protective factors included breastfeeding, healthy eating, decreased intake of energy-dense unhealthy foods, physical activity, and infants reaching developmental milestones, such as walking (32, 42, 58, 60, 82, 89).

Dental attendance was not consistently seen as a protective factor against ECC. Many parents considered accessing dental care only for symptom treatment (36, 45, 48, 49, 53, 54, 81, 94, 97, 98, 102, 104, 114, 120–122). Parents who valued preventative dental care may only do so after negative experiences of ECC from their families and communities (52, 54, 56, 94, 97, 111).

Genetic susceptibility to ECC or higher body weight was frequently discussed as being intrinsic. Therefore, ECC, overweight, or obesity was inevitable or untreatable, even with preventative behaviours, such as regular dental attendance or lifestyle behaviours (33, 35, 38, 39, 42, 43, 46–51, 57, 60, 61, 65, 66, 68, 69, 71, 77, 86–89, 94, 95, 98, 99, 105, 112, 115, 118, 123).

Some beliefs reflect unclear understanding of biological causes of ECC or overweight and obesity. This included ECC being caused by poor tooth formation from undernutrition, e.g., insufficient milk or calcium during pregnancy or infancy (46, 55, 95, 103, 112, 118), or use of antibiotics (49, 110); and overweight and obesity being caused by metabolism, poor sleep, stress from bullying, and hormones in animal production (35, 60, 65–67, 77). Parents also believed mouth washing was sufficient for oral hygiene, especially when feeding to sleep made tooth-brushing difficult (36, 76, 91). For parents who misunderstood the causes of ECC, cariogenic risk factors could be maintained—for example, parents who believed that chewing or sucking of bottle teats caused ECC (46, 95, 117, 118) replaced bottles with cups during feeding to sleep.

The cariogenicity and obesogenicity of foods and drinks could be poorly understood, as demonstrated through beliefs on: the healthiness of fruit juice, fruit drink and soft drinks; dilution of sugary drinks with water being protective against ECC; or use of unrefined sugars, such as jaggery or honey (49, 52, 54, 60, 61, 85, 91, 92, 94, 95, 112, 117, 120). Some parents were unaware that certain foods or drinks—such as candy, chocolate, cookies, potato chips, or ice cream—were cariogenic or obesogenic (49, 72, 81, 91, 95) or felt that certain foods, while cariogenic, remained nourishing (36, 54, 81, 94).

#### 3.7.4 The impact of unhealthy teeth and weight for children

For parents, unhealthy teeth and weight was defined by consequential impacts. Understanding the life course trajectory of overweight, obesity and ECC could inform how parents responded to these conditions. However, there were notable differences in perceptions on ECC, compared to overweight and obesity. Fewer parents proactively discussed prevention, while those who did could be informed by experiences in their family and community, such as their childhood or the care of other children (38, 39, 41, 45, 75, 82, 84, 85, 94–96, 101, 107).

The consequences of ECC related to the permanence of primary and permanent teeth. Parents reacted to poor health in primary teeth when it impacted nutrition, speaking, child mood and behaviour, or when it was believed to impact the health of permanent teeth (36, 47, 48, 51–53, 94, 99, 100, 114). While asymptomatic ECC, such as white spots, did not prompt health seeking by some parents, parents also reported being dismissed and unsupported by health professionals when seeking advice on asymptomatic ECC (36, 46, 48, 111, 112, 114). Parents' views on primary teeth was related to its impermanence, and varied from apathy to concern (13, 36, 44, 47–50, 53, 56, 94, 98, 102, 113, 121). For some parents, the health of primary teeth appeared unrelated to the health of permanent teeth, and primary teeth were “practice” for oral hygiene behaviours for permanent teeth (13, 48, 49, 53, 111). Subsequently, dental care reflected a focus on acute symptom treatment, such distress and poor behaviour from dental pain, with minimal focus on ECC prevention (94, 112, 114, 124). Longer-term dental care behaviours varied after experiences of ECC: for some, the resolution of symptoms after removal of decayed teeth led to dental care behaviours not being maintained, while others were motivated to maintain dental care behaviours (49, 94, 95, 99, 101, 106, 110, 111).

Consistent with this, overweight and obesity—either as a present diagnosis or a future risk—was not seen as significant if children appeared asymptomatic (40, 43, 67, 84, 89, 125). Overweight or obesity was detrimental when it was consequential, such as parental back pain when carrying a heavy infant; physical incapacity, including delays in reaching developmental milestones, shortness of breath, and tiredness during play; and teasing or bullying that impacted children's mental health and self-esteem (32–35, 37–39, 42, 43, 60, 61, 64–67, 77, 82–84, 87–89, 96, 116).

Understanding the life course trajectory of overweight or obesity meant that some parents anticipated the risk of future weight-related health problems and the normalization of obesogenic lifestyle behaviours into adulthood (32, 33, 37, 39, 43, 60, 64–66, 75, 77, 82, 84, 85, 87–89). However, despite significant health consequences, overweight or obesity were unimportant at *present*, and would only be a problem beyond infancy or school age (58, 65, 68, 69, 78, 87). Subsequently, parents believed that obesogenic behaviours could be addressed later, or that body weight would self-resolve when child height or physical activity intrinsically increased (58, 64–66, 69, 83, 84, 87, 89, 116).

### 3.8 Theme 3: supporting parents to maintain their child's health

While there were distinctions in framing their needs for promoting children's oral health and healthy eating, and preventing ECC, overweight or obesity, parents' need for information and resources was consistent. Parents discussed their ability to successfully support healthy teeth and eating being based on internal and external loci of control.

#### 3.8.1 Information sources and services utilised by parents

Parents utilised professional and non-professional sources for information about children's health, such as medical, nursing, dentistry, allied health professionals and government programs, and family, friends, peers and advertising, respectively. Teachers, early childhood educators and social workers were also important non-health professional sources of information (45, 46, 49, 55, 76, 88, 97, 101, 103, 104, 108, 117, 126). Attitudes towards these sources were dependent on being aligned with parents’ expectations. Parents' satisfaction with professional sources was impacted by expectations of care, agreement with information received, comprehensiveness of education, quality of services, and the parent-provider relationship, particularly a clinician's competence when working with children (13, 32, 35, 38, 49, 53, 58, 60, 63, 75–78, 81, 82, 84, 87, 89, 90, 92, 94, 97–99, 103, 107, 111, 113, 114, 120, 122, 123, 126). The use of non-professional sources of information varied: many parents trusted the advice and experiences of other parents and family members (13, 35–37, 46, 48, 58, 76, 83, 87, 92, 93, 96), while few parents felt this information did not align with professional advice (74, 87, 99, 114). Notably, information from health professionals could impact how parents perceived the severity or significance of ECC, overweight or obesity (32, 46, 48, 58, 65, 84, 86, 89, 112) – and parents were confused when received conflicting information from trusted sources or were advised by health professionals that further health care seeking was not needed (35, 53, 54, 76, 84, 89, 99, 111, 114, 121, 125).

Parents reported difficulty accessing health providers being a barrier to accessing information or care to support their child's health. This included actual or perceived cost of services; time barriers; inability to receive specialist referrals; attendance to service locations, related to transport challenges and isolation of rural, regional and remote areas; language barriers; and refusal of dental services for children reportedly too young for care (13, 43–46, 50, 52–54, 56, 81, 82, 94, 98–100, 111–114, 117, 120).

#### 3.8.2 Resources needed for supporting healthy teeth and weight

Parents identified information and resource needs based on gaps in care, which may indicate that routine well-child services were not meeting parent needs, or that parents were not accessing health services. These topics included breastfeeding and introduction of solid foods, healthy eating, physical activity, sleep, understanding growth charts, oral health and care, and general parenting, such as responding to infant cues, and child development. Information also needed to be specific, factual and practical (32–34, 47, 50, 53, 58, 62–64, 70, 74, 76, 78, 80–84, 87, 88, 90, 96, 102, 103, 108, 111–113, 115–117, 120, 125, 126).

Parents offered suggestions on community- or school-based programs that could be available, such as parent groups, playgroups, education programs with peer learning, dental outreach, and activity programs (33, 48, 50, 53, 58, 80, 88, 96, 109, 125). To be accessible, these programs needed to be free, provide childcare, and involve children in age-appropriate education (34, 125). Resources also needed to be proactive instead of reactive, such as the establishment of food assistance programs or food co-ops with healthy food, and the provision of dental care supplies, like toothbrushes and toothpaste, through community services (34, 45, 50, 87, 100, 108).

Parents wanted improved access to dental care for children, such as affordable, subsidised or free dental care; alignment of dental services with medical services; or access to paediatric dentists (45, 53, 54, 98, 99, 106, 108, 113). While free or subsidized dental care could be available in countries with public healthcare, this was not broadly known, or was perceived as poorer quality compared to private health services (44, 51, 53, 54).

Resources were needed to meet diverse language needs, such as low literacy resources, access to interpreters, and increased language diversity (38, 44, 48, 53, 63, 87, 90, 102, 113). Resources in different formats or modes were required, varying from print resources; digital resources, such as electronic newsletters, internet and social media websites; and in-person resources, such as parenting classes, and community health workers to link parents to services (44, 48, 63, 87, 90, 120, 125).

#### 3.8.3 Supporting parental self-efficacy

Parents identified practices to raise a healthy child in theme 1.2. However, internal and external factors limit their efficacy in doing this.

While parents considered tooth brushing as important, they experienced barriers, including self-efficacy, lack of time, and responding to a resistant child (13, 36, 48–50, 53, 80, 81, 94, 95, 97, 99–105, 110, 122). Some parents reported teeth cleaning that was not best-practice, such as unsupervised tooth brushing before 7 years of age, mouth rinsing or cleaning with cloth instead of tooth brushing, delayed tooth brushing initiation, or suboptimal tooth brushing time (44, 49, 53, 97, 101, 103, 110, 119, 120, 122).

Parents recognised the difficulty in supporting children's healthy eating in obesogenic and cariogenic environments. Parents felt their attempts to encourage healthy eating were undermined by other carers and children's peers (13, 37, 42, 44, 49, 50, 53, 56, 60, 61, 64, 66, 67, 78, 81–84, 93, 99, 104, 111, 115, 127). Similar to tooth brushing, parents experienced barriers to providing healthy foods, including self-efficacy, children's food preferences, financial and time costs of providing healthy food, and the convenience and inexpensive nature of unhealthy foods (13, 34, 35, 50, 60–62, 66, 74, 77, 81, 82, 84, 85, 88, 96, 99, 100, 116, 117, 125, 126).

Parents' use of food, or response to signs of hunger, indicated these were barriers to healthy eating. Parents reported the use of persuasive feeding to pressure children into eating healthy foods, reduce disruptive behaviour, or to reward good behaviour (13, 37, 38, 42, 49, 57, 64, 75, 77, 81, 83, 85, 87, 126). Parents felt unable to deny food requests, challenge food refusal, or address resistance from children, even during food introduction for infants (35, 36, 38, 44, 49, 61, 64, 71, 75, 83, 85, 91–94, 97, 98, 115, 123). This awareness of foods being unhealthy while also feeling children *could not* be refused is distinct from theme 1.2.2, where food *should not* be denied and should be provided to encourage diverse food experiences.

Ensuring infants and children were fed, particularly “fussy” or “picky” eaters, could lead to prolonged bottle feeding (36, 42, 49, 90). Likewise, parents' concerns that children's refusal to use drinking cups or eat healthy foods would cause undernutrition led to encouragement or maintenance of cariogenic or obesogenic feeding behaviours (35, 49, 61, 76, 81, 90, 93, 95, 97, 99). Parents did not feel confident to deny food for children who reported being hungry (32, 35, 42, 60, 61, 97, 109, 115), which was similar to parents feeling that feeding to soothe was the only behaviour that could settle crying, tantrums or fussiness (13, 57, 64, 80, 123).

## 4 Discussion

This qualitative systematic review of 98 publications centres the lived experiences of parents of infants and children, which focus on valuing child wellness, and responding to unwellness when unhealthy weight or ECC symptoms were experienced. Parents valued messages about healthy weight and teeth, but not messages about preventing obesity or ECC. Parents had reactive responses to overweight, obesity or ECC – such as dental attendance for ECC treatment but not routine check-ups for prevention, or limiting obesogenic foods after overweight or obesity was clinically diagnosed. Parents reported information needs from professional sources on nutrition, dental care and responsive parenting, which may indicate gaps in well-child visits where this information is typically provided.

These review findings are consistent with previous qualitative research, where there was poor understanding of cariogenic risk from formula, bottle or night-time feeding; the addition of foods into bottles being obesogenic and cariogenic; cultural preferences for larger or heavier infants and children; and low self-efficacy in tooth brushing or responsive child feeding (128–131). This is congruent with questionnaires which demonstrate varying levels of parental knowledge on bottle use and preventative dental care on ECC risk (132–134) – however, knowledge of bottle use may not affect ECC status or use of obesogenic feeding behaviours (134, 135). Key messages on formula and bottle feeding as causal factors of obesity and ECC were not recalled: parents were largely unaware of the association, with some only becoming aware after unhealthy weight gain or ECC occurred (13, 36, 48, 49, 53, 76, 86, 94, 95, 117, 118); and few were aware that frequency of feeding could result in overnutrition or sugar exposure as obesity and ECC risk factors (75, 97, 105). Community and primary health care-based interventions for parents can improve bottle use behaviours and routine preventative dental care (11, 133, 136–138), which emphasises the importance of education and support on dental care and nutrition throughout pregnancy and early childhood.

These findings indicate the need for communication that address parent priorities and support self-efficacy. This has implications for health messages and behaviour change technique-informed strategies in program design, such as strength-based messages (i.e., healthy teeth and growth) instead of messages about obesity or ECC prevention. Healthy teeth were prioritised for aesthetic appearance, including attractive smiles, while the function of teeth for eating was only discussed as a consequence of ECC. In contrast, healthy weight was prioritised for the aesthetics of size demonstrating adequate growth, and the function of weight reflecting good dietary intake and ability to play. Impairments in these, along with bullying or teasing, indicated children at an unhealthy weight. These differences were also seen when interpreting the life-course trajectory of ECC, overweight or obesity. While parents could identify the immediate consequences of ECC, few identified the long-term risk of dental caries in permanent teeth. Conversely, while parents could identify chronic conditions associated with unhealthy weight, these were considered to be relevant only if overweight or obesity occurred beyond infancy and childhood. Even where parents could identify cariogenic or obesogenic risk factors, they experienced challenges in health-promoting behaviours and ceasing cariogenic or obesogenic risk behaviours, such as settling child crying with feeding. Responsive parenting supports parents to interact with infants and children to develop child regulation and autonomy—the INSIGHT trial demonstrated improved maternal structured feeding practices (139) and modestly improved child weight outcomes at 3 years age (140), while the Sleep Strong African American Families trial demonstrated improvements in maternal responsive feeding (141) and reduced odds of infant rapid weight gain at 16 weeks age (142). Communication to parents should include: strength-based messaging and strategies to build parental self-efficacy; emphasize the relevance of health-promoting behaviours from infancy; and identify how rapidly ECC and overweight or obesity can occur, even in infancy and childhood.

These findings also indicate resource needs and the potential for co-design in program delivery. Parents identified the use or need for community- or school-based resources; distribution of dental health products, like toothbrushes or toothpaste; and education, workshops or health screening in schools and community venues. Specific focus was given towards affordable access to dental care, including services for children, by participants internationally and across socioeconomic statuses. Financial accessibility for medical treatment, particularly dental care, require health service restructure and may not be feasible to address without system-level change—however, strategies to increase access to care may include public health messaging about dental service eligibility, which may not be widely known (94), and preventative care delivery or home visiting with community health workers (143–146). Many articles in this review (86 of 98) involved vulnerable populations with social disadvantage or changing food environments during economic transition—therefore, co-design and community partnership can support equity in program reach and address upstream social determinants of health (147). Community stakeholder involvement in program design may support uptake and sustainability, such as the currently ongoing Healthy Smile Happy Child dental health program (148) and Whanau Pakari early childhood obesity program (149).

Experiences recalled by parents also indicate the need for changes in health provider practice. Parents reported confusion or frustration from health providers who dismissed or normalised obesity or ECC diagnosis or symptoms (84, 111, 114); refused to provide referrals for allied health or dental care (82, 111); or would not provide dental services for children under three years age (111, 121). This demonstrates the need for clear dissemination of up-to-date practice guidelines, which encourage dental attendance at one year age to establish preventative health behaviours and routine check-ups (150, 151), as well as continuing practice development for providers on early identification of obesity and ECC, such as using growth charts to identify unhealthy weight gain or addressing ECC from white spot identification (152, 153). Targeted or tailored messages and services by community health workers, especially for high-risk populations, may increase dental service uptake (143, 154). There are opportunities to integrate oral health into primary health care by non-dental practitioners (155) or oral health practitioners to discuss obesity prevention (156, 157), especially where obesogenic and cariogenic behaviours overlap. A common risk factor approach to obesity and ECC, across child health professional roles, can be supported with inclusion in tertiary education curricula, interdisciplinary collaboration, continuing practice development, and use of best-practice resources—such as screening tools and client-facing resources—to undertake screening, behaviour change counselling, and referral to services (157–159).

Quality appraisal with the CASP Qualitative Checklist indicated that articles tended to have good methodological quality. All articles had clear research aims and appropriate use of qualitative research methods. Many articles had clearly stated findings, with results linked to current practice, transferability to other populations or relevance to further areas of research. However, there could be unclear or high risk of bias in not explicitly reporting the researcher-participant relationship, details of informed consent or explanation of the research process for participants, or rigorous data analysis processes, as these aspects tended by briefly or not reported. As the use of social science research expands in health disciplines, it is important that future research addresses rigorous qualitative research methodology, and reflexivity and power relations between researchers and participants (17).

This review has several strengths. First, it integrates research from separate health disciplines for two conditions that share overlapping aetiologies—notably, a common finding when discussing both obesity and ECC was low parental self-efficacy to respond to children's unsettled behaviour and to address unhealthy feeding behaviours by other carers. Second, the findings of the included studies were broad, with 98 articles identified from nearly 6,000 references, which indicate a comprehensive search. Many qualitative studies on overweight, obesity or obesogenic behaviours in early childhood were excluded due to lack of focus on body weight or size. However, this refined the focus of this review as the body of literature on paediatric obesity is extensive and the inclusion of further studies would not have expanded findings. Third, the use of reflexive thematic analysis centred the parental perspective, particularly on child feeding, teeth and weight, and why cariogenic and obesogenic feeding behaviours may be valued. This, in turn, can inform program design which addresses community values or uses a strength-based approach to support behaviour change.

Limitations of this review relate to techniques to enhance trustworthiness and credibility of data analysis. One reviewer, HC, undertook database searching; study screening for inclusion and exclusion; coding in NVivo; and generation of the primary findings using thematic analysis. Study screening, NVivo coding and thematic analysis in duplicate would improve credibility of findings. However, inter-rater reliability was undertaken with a second reviewer to confirm full-text studies included for analysis; compare coding; and compare critical appraisal of qualitative methodology—the inter-rater reliability between reviewers was high and indicate that the work undertaken by HC is trustworthy and credible. Further, final themes and subthemes were finalised with discussion across co-authors. Diverse populations were included in this review, with 50 of 77 studies from Western countries (United States, Australia, Canada, New Zealand, western Europe) involving cultural minorities or Indigenous populations, and the remaining 21 studies including cultural majority populations across South America, Mexico and Asia. While preferences for larger or heavier child body size of weight cannot be generalisable across diverse populations, this perspective was observed across cultural groups in 13 included studies—and similar perspectives are demonstrated towards the lack of importance of primary teeth compared to permanent teeth by participants across different cultural groups in 13 included studies. However, few included studies involved populations or diaspora from African countries (one study, South Africa (43); one study, West Indies (53); Black American or British parents, 15 studies (35, 39, 40, 42, 57, 70, 72, 78, 79, 87, 89, 102, 111, 117, 121); migrants from African countries and the Caribbean (37, 44, 48–50, 58, 63, 113, 127), 9 studies), indicating underrepresentation in research.

This systematic qualitative review of 98 articles suggests the need for a holistic approach to prevention of obesity and dental caries in early childhood, and to address overlapping dietary behaviours. By using a focus on parents' lived experience, it identifies the need for strength-based communication and opportunities to improve understanding of formula and bottle feeding as cariogenic and obesogenic risk factors. Findings also demonstrate the need for continuing practice development for health professionals to identify and address early symptoms of obesity and ECC, including referral to dental care, and holistic approaches to dental and obesity prevention care by primary care and oral health practitioners.

Understanding these parent perspectives on healthy teeth and weight in early childhood will support the development of health promotion initiatives, including education resources and messages to support behaviour change. These findings will inform community stakeholder engagement, including focus groups and co-design workshops to develop an obesity and ECC prevention intervention through changing formula and bottle feeding behaviours.

## 5 Conclusion

This qualitative systematic review centred the perspectives of parents on overweight, obesity and dental caries in early childhood. Parents are focused on child wellness and value messages on healthy teeth and weight. Parents react to health conditions, such as ECC or obesity, once children experience symptoms that interfere with wellbeing—however, they may not undertake preventative behaviours, such as routine dental check-ups, or limiting obesogenic foods before overweight or obesity is clinically diagnosed. Parents identified information needs that highlight the importance of supporting parental self-efficacy for tooth brushing, healthy eating and responsive parenting. These findings also highlight the importance of health providers in early identification of ECC and obesity, and provision of accurate, tailored and up-to-date information and advice.

## Data Availability

The data presented in this study can be found in online repositories. The names of the repository/repositories and accession number(s) can be found in the article/Supplementary Material.
