# Supplementary material for: Carer perspectives on overweight, obesity and dental caries in early childhood: findings from a systematic qualitative review
Source: Front Oral Health. 2025 Jun 18;6:1524715. doi: 10.3389/froh.2025.1524715 (PMC12213562; doi:10.3389/froh.2025.1524715)
Supplement: Supplementary file 2 [file Table2.docx]

Supplementary Files 2 to 7

# Supplementary Table 2. Database search: Medline, dental caries.

| **#** | **Searches** | **Results** |
| --- | --- | --- |
| 1 | Infant/ | 820099 |
| 2 | Infant, Newborn/ | 626947 |
| 3 | Child/ | 1763793 |
| 4 | 1 or 2 or 3 | 2454427 |
| 5 | Dental Caries/ | 46946 |
| 6 | "tooth decay".mp. | 1587 |
| 7 | dental care.mp. or Dental Care/ | 40048 |
| 8 | "dental caries".mp. | 55924 |
| 9 | "early childhood caries".mp. | 2009 |
| 10 | 5 or 6 or 7 or 8 or 9 | 93637 |
| 11 | Health Knowledge, Attitudes, Practice/ | 118834 |
| 12 | Culture/ | 33647 |
| 13 | Health Education/ | 62045 |
| 14 | Attitude to Health/ | 84906 |
| 15 | Health Behavior/ | 53122 |
| 16 | knowledge.mp. | 694688 |
| 17 | belief.mp. | 29861 |
| 18 | perception.mp. | 352475 |
| 19 | perspective.mp. | 186882 |
| 20 | 11 or 12 or 13 or 14 or 15 or 16 or 17 or 18 or 19 | 1367774 |
| 21 | Parents/ | 69094 |
| 22 | parent.mp. | 144873 |
| 23 | Caregivers/ | 41043 |
| 24 | caregiver.mp. | 26194 |
| 25 | carer.mp. | 4631 |
| 26 | guardian.mp. or Legal Guardians/ | 4683 |
| 27 | 21 or 22 or 23 or 24 or 25 or 26 | 241408 |
| 28 | 4 and 10 and 20 and 27 | 588 |
| 29 | Qualitative Research/ or qualitative.mp. | 227947 |
| 30 | Interview/ or interview.mp. | 151596 |
| 31 | "focus group".mp. or Focus Groups/ | 52044 |
| 32 | 29 or 30 or 31 | 500179 |
| 33 | 28 and 32 | 96 |

# Supplementary Table 3. Database search: CINAHL, dental caries.

| [Search ID#](javascript:__doPostBack('ctl00$ctl00$FindField$FindField$historyControl$ReorderHistoryLink','')) | **Search Terms** | **Actions** |
| --- | --- | --- |
| S36 | S30 AND S35 | [View Results](javascript:__doPostBack('ctl00$ctl00$FindField$FindField$historyControl$HistoryRepeater$ctl00$linkResults','')) (131)  [View Details](javascript:showShDetails(%22ctl00_ctl00_FindField_FindField_historyControl_ctrlPopup%22,%20%22S37%22);)  [Edit](https://web-s-ebscohost-com.ezproxy.library.sydney.edu.au/Legacy/Views/UserControls/EHOST/) |
| S35 | S31 OR S32 OR S33 OR S34 | [View Results](javascript:__doPostBack('ctl00$ctl00$FindField$FindField$historyControl$HistoryRepeater$ctl01$linkResults','')) (234,571)  [View Details](javascript:showShDetails(%22ctl00_ctl00_FindField_FindField_historyControl_ctrlPopup%22,%20%22S36%22);)  [Edit](https://web-s-ebscohost-com.ezproxy.library.sydney.edu.au/Legacy/Views/UserControls/EHOST/) |
| S34 | (MH "Focus Groups") | [View Results](javascript:__doPostBack('ctl00$ctl00$FindField$FindField$historyControl$HistoryRepeater$ctl03$linkResults','')) (47,530)  [View Details](javascript:showShDetails(%22ctl00_ctl00_FindField_FindField_historyControl_ctrlPopup%22,%20%22S34%22);)  [Edit](https://web-s-ebscohost-com.ezproxy.library.sydney.edu.au/Legacy/Views/UserControls/EHOST/) |
| S33 | (MH "Semi-Structured Interview") OR (MH "Structured Interview") | [View Results](javascript:__doPostBack('ctl00$ctl00$FindField$FindField$historyControl$HistoryRepeater$ctl04$linkResults','')) (81,317)  [View Details](javascript:showShDetails(%22ctl00_ctl00_FindField_FindField_historyControl_ctrlPopup%22,%20%22S33%22);)  [Edit](https://web-s-ebscohost-com.ezproxy.library.sydney.edu.au/Legacy/Views/UserControls/EHOST/) |
| S32 | TX qualitative research or qualitative study or qualitative methods or interview | [View Results](javascript:__doPostBack('ctl00$ctl00$FindField$FindField$historyControl$HistoryRepeater$ctl05$linkResults','')) (579,848)  [View Details](javascript:showShDetails(%22ctl00_ctl00_FindField_FindField_historyControl_ctrlPopup%22,%20%22S32%22);)  [Edit](https://web-s-ebscohost-com.ezproxy.library.sydney.edu.au/Legacy/Views/UserControls/EHOST/) |
| S31 | (MH "Qualitative Studies+") | [View Results](javascript:__doPostBack('ctl00$ctl00$FindField$FindField$historyControl$HistoryRepeater$ctl06$linkResults','')) (165,514)  [View Details](javascript:showShDetails(%22ctl00_ctl00_FindField_FindField_historyControl_ctrlPopup%22,%20%22S31%22);)  [Edit](https://web-s-ebscohost-com.ezproxy.library.sydney.edu.au/Legacy/Views/UserControls/EHOST/) |
| S30 | S6 AND S11 AND S23 AND S29 | [View Results](javascript:__doPostBack('ctl00$ctl00$FindField$FindField$historyControl$HistoryRepeater$ctl07$linkResults','')) (3,738)  [View Details](javascript:showShDetails(%22ctl00_ctl00_FindField_FindField_historyControl_ctrlPopup%22,%20%22S30%22);)  [Edit](https://web-s-ebscohost-com.ezproxy.library.sydney.edu.au/Legacy/Views/UserControls/EHOST/) |
| S29 | S24 OR S25 OR S26 OR S27 OR S28 | [View Results](javascript:__doPostBack('ctl00$ctl00$FindField$FindField$historyControl$HistoryRepeater$ctl08$linkResults','')) (1,235,323)  [View Details](javascript:showShDetails(%22ctl00_ctl00_FindField_FindField_historyControl_ctrlPopup%22,%20%22S29%22);)  [Edit](https://web-s-ebscohost-com.ezproxy.library.sydney.edu.au/Legacy/Views/UserControls/EHOST/) |
| S28 | TX ( parents or caregivers or mother or father or parent or family ) OR TX guardians OR TX carers | [View Results](javascript:__doPostBack('ctl00$ctl00$FindField$FindField$historyControl$HistoryRepeater$ctl09$linkResults','')) (1,233,980)  [View Details](javascript:showShDetails(%22ctl00_ctl00_FindField_FindField_historyControl_ctrlPopup%22,%20%22S28%22);)  [Edit](https://web-s-ebscohost-com.ezproxy.library.sydney.edu.au/Legacy/Views/UserControls/EHOST/) |
| S27 | (MH "Guardianship, Legal+") | [View Results](javascript:__doPostBack('ctl00$ctl00$FindField$FindField$historyControl$HistoryRepeater$ctl10$linkResults','')) (1,877)  [View Details](javascript:showShDetails(%22ctl00_ctl00_FindField_FindField_historyControl_ctrlPopup%22,%20%22S27%22);)  [Edit](https://web-s-ebscohost-com.ezproxy.library.sydney.edu.au/Legacy/Views/UserControls/EHOST/) |
| S26 | (MH "Caregivers") | [View Results](javascript:__doPostBack('ctl00$ctl00$FindField$FindField$historyControl$HistoryRepeater$ctl11$linkResults','')) (39,397)  [View Details](javascript:showShDetails(%22ctl00_ctl00_FindField_FindField_historyControl_ctrlPopup%22,%20%22S26%22);)  [Edit](https://web-s-ebscohost-com.ezproxy.library.sydney.edu.au/Legacy/Views/UserControls/EHOST/) |
| S25 | (MH "Foster Parents") | [View Results](javascript:__doPostBack('ctl00$ctl00$FindField$FindField$historyControl$HistoryRepeater$ctl12$linkResults','')) (1,045)  [View Details](javascript:showShDetails(%22ctl00_ctl00_FindField_FindField_historyControl_ctrlPopup%22,%20%22S25%22);)  [Edit](https://web-s-ebscohost-com.ezproxy.library.sydney.edu.au/Legacy/Views/UserControls/EHOST/) |
| S24 | (MH "Parents+") | [View Results](javascript:__doPostBack('ctl00$ctl00$FindField$FindField$historyControl$HistoryRepeater$ctl13$linkResults','')) (103,443)  [View Details](javascript:showShDetails(%22ctl00_ctl00_FindField_FindField_historyControl_ctrlPopup%22,%20%22S24%22);)  [Edit](https://web-s-ebscohost-com.ezproxy.library.sydney.edu.au/Legacy/Views/UserControls/EHOST/) |
| S23 | S12 OR S13 OR S14 OR S15 OR S16 OR S17 OR S18 OR S19 OR S20 OR S21 OR S22 | [View Results](javascript:__doPostBack('ctl00$ctl00$FindField$FindField$historyControl$HistoryRepeater$ctl14$linkResults','')) (2,444,116)  [View Details](javascript:showShDetails(%22ctl00_ctl00_FindField_FindField_historyControl_ctrlPopup%22,%20%22S23%22);)  [Edit](https://web-s-ebscohost-com.ezproxy.library.sydney.edu.au/Legacy/Views/UserControls/EHOST/) |
| S22 | TX perspective or perception or opinion or experience or attitude | [View Results](javascript:__doPostBack('ctl00$ctl00$FindField$FindField$historyControl$HistoryRepeater$ctl15$linkResults','')) (1,639,355)  [View Details](javascript:showShDetails(%22ctl00_ctl00_FindField_FindField_historyControl_ctrlPopup%22,%20%22S22%22);)  [Edit](https://web-s-ebscohost-com.ezproxy.library.sydney.edu.au/Legacy/Views/UserControls/EHOST/) |
| S21 | (MH "Health Behavior+") | [View Results](javascript:__doPostBack('ctl00$ctl00$FindField$FindField$historyControl$HistoryRepeater$ctl16$linkResults','')) (112,270)  [View Details](javascript:showShDetails(%22ctl00_ctl00_FindField_FindField_historyControl_ctrlPopup%22,%20%22S21%22);)  [Edit](https://web-s-ebscohost-com.ezproxy.library.sydney.edu.au/Legacy/Views/UserControls/EHOST/) |
| S20 | TX attitudes or perceptions or opinions or thoughts or feelings or beliefs | [View Results](javascript:__doPostBack('ctl00$ctl00$FindField$FindField$historyControl$HistoryRepeater$ctl17$linkResults','')) (1,181,453)  [View Details](javascript:showShDetails(%22ctl00_ctl00_FindField_FindField_historyControl_ctrlPopup%22,%20%22S20%22);)  [Edit](https://web-s-ebscohost-com.ezproxy.library.sydney.edu.au/Legacy/Views/UserControls/EHOST/) |
| S19 | (MH "Attitude to Health+") | [View Results](javascript:__doPostBack('ctl00$ctl00$FindField$FindField$historyControl$HistoryRepeater$ctl18$linkResults','')) (171,457)  [View Details](javascript:showShDetails(%22ctl00_ctl00_FindField_FindField_historyControl_ctrlPopup%22,%20%22S19%22);)  [Edit](https://web-s-ebscohost-com.ezproxy.library.sydney.edu.au/Legacy/Views/UserControls/EHOST/) |
| S18 | (MH "Health Education+") | [View Results](javascript:__doPostBack('ctl00$ctl00$FindField$FindField$historyControl$HistoryRepeater$ctl19$linkResults','')) (137,535)  [View Details](javascript:showShDetails(%22ctl00_ctl00_FindField_FindField_historyControl_ctrlPopup%22,%20%22S18%22);)  [Edit](https://web-s-ebscohost-com.ezproxy.library.sydney.edu.au/Legacy/Views/UserControls/EHOST/) |
| S17 | TX beliefs or perceptions or views or attitudes or opinions or knowledge | [View Results](javascript:__doPostBack('ctl00$ctl00$FindField$FindField$historyControl$HistoryRepeater$ctl20$linkResults','')) (1,517,587)  [View Details](javascript:showShDetails(%22ctl00_ctl00_FindField_FindField_historyControl_ctrlPopup%22,%20%22S17%22);)  [Edit](https://web-s-ebscohost-com.ezproxy.library.sydney.edu.au/Legacy/Views/UserControls/EHOST/) |
| S16 | (MH "Attitude to Illness+") | [View Results](javascript:__doPostBack('ctl00$ctl00$FindField$FindField$historyControl$HistoryRepeater$ctl21$linkResults','')) (24,419)  [View Details](javascript:showShDetails(%22ctl00_ctl00_FindField_FindField_historyControl_ctrlPopup%22,%20%22S16%22);)  [Edit](https://web-s-ebscohost-com.ezproxy.library.sydney.edu.au/Legacy/Views/UserControls/EHOST/) |
| S15 | (MH "Health Beliefs") | [View Results](javascript:__doPostBack('ctl00$ctl00$FindField$FindField$historyControl$HistoryRepeater$ctl22$linkResults','')) (14,694)  [View Details](javascript:showShDetails(%22ctl00_ctl00_FindField_FindField_historyControl_ctrlPopup%22,%20%22S15%22);)  [Edit](https://web-s-ebscohost-com.ezproxy.library.sydney.edu.au/Legacy/Views/UserControls/EHOST/) |
| S14 | (MH "Culture+") | [View Results](javascript:__doPostBack('ctl00$ctl00$FindField$FindField$historyControl$HistoryRepeater$ctl23$linkResults','')) (200,925)  [View Details](javascript:showShDetails(%22ctl00_ctl00_FindField_FindField_historyControl_ctrlPopup%22,%20%22S14%22);)  [Edit](https://web-s-ebscohost-com.ezproxy.library.sydney.edu.au/Legacy/Views/UserControls/EHOST/) |
| S13 | TX health knowledge or health information or health understanding | [View Results](javascript:__doPostBack('ctl00$ctl00$FindField$FindField$historyControl$HistoryRepeater$ctl24$linkResults','')) (281,606)  [View Details](javascript:showShDetails(%22ctl00_ctl00_FindField_FindField_historyControl_ctrlPopup%22,%20%22S13%22);)  [Edit](https://web-s-ebscohost-com.ezproxy.library.sydney.edu.au/Legacy/Views/UserControls/EHOST/) |
| S12 | TX health knowledge, attitudes, practice or knowledge, attitudes, practice | [View Results](javascript:__doPostBack('ctl00$ctl00$FindField$FindField$historyControl$HistoryRepeater$ctl25$linkResults','')) (10,670)  [View Details](javascript:showShDetails(%22ctl00_ctl00_FindField_FindField_historyControl_ctrlPopup%22,%20%22S12%22);)  [Edit](https://web-s-ebscohost-com.ezproxy.library.sydney.edu.au/Legacy/Views/UserControls/EHOST/) |
| S11 | S7 OR S8 OR S9 OR S10 | [View Results](javascript:__doPostBack('ctl00$ctl00$FindField$FindField$historyControl$HistoryRepeater$ctl26$linkResults','')) (34,455)  [View Details](javascript:showShDetails(%22ctl00_ctl00_FindField_FindField_historyControl_ctrlPopup%22,%20%22S11%22);)  [Edit](https://web-s-ebscohost-com.ezproxy.library.sydney.edu.au/Legacy/Views/UserControls/EHOST/) |
| S10 | (MH "Dental Care+") OR (MH "Dental Care for Children") | [View Results](javascript:__doPostBack('ctl00$ctl00$FindField$FindField$historyControl$HistoryRepeater$ctl27$linkResults','')) (17,104)  [View Details](javascript:showShDetails(%22ctl00_ctl00_FindField_FindField_historyControl_ctrlPopup%22,%20%22S10%22);)  [Edit](https://web-s-ebscohost-com.ezproxy.library.sydney.edu.au/Legacy/Views/UserControls/EHOST/) |
| S9 | "early childhood caries" | [View Results](javascript:__doPostBack('ctl00$ctl00$FindField$FindField$historyControl$HistoryRepeater$ctl28$linkResults','')) (932)  [View Details](javascript:showShDetails(%22ctl00_ctl00_FindField_FindField_historyControl_ctrlPopup%22,%20%22S9%22);)  [Edit](https://web-s-ebscohost-com.ezproxy.library.sydney.edu.au/Legacy/Views/UserControls/EHOST/) |
| S8 | TX dental caries or dental decay or dental cavity or dental cavities or tooth decay | [View Results](javascript:__doPostBack('ctl00$ctl00$FindField$FindField$historyControl$HistoryRepeater$ctl29$linkResults','')) (19,937)  [View Details](javascript:showShDetails(%22ctl00_ctl00_FindField_FindField_historyControl_ctrlPopup%22,%20%22S8%22);)  [Edit](https://web-s-ebscohost-com.ezproxy.library.sydney.edu.au/Legacy/Views/UserControls/EHOST/) |
| S7 | (MH "Dental Caries") | [View Results](javascript:__doPostBack('ctl00$ctl00$FindField$FindField$historyControl$HistoryRepeater$ctl30$linkResults','')) (13,280)  [View Details](javascript:showShDetails(%22ctl00_ctl00_FindField_FindField_historyControl_ctrlPopup%22,%20%22S7%22);)  [Edit](https://web-s-ebscohost-com.ezproxy.library.sydney.edu.au/Legacy/Views/UserControls/EHOST/) |
| S6 | S1 OR S2 OR S3 OR S4 OR S5 | [View Results](javascript:__doPostBack('ctl00$ctl00$FindField$FindField$historyControl$HistoryRepeater$ctl31$linkResults','')) (949,421)  [View Details](javascript:showShDetails(%22ctl00_ctl00_FindField_FindField_historyControl_ctrlPopup%22,%20%22S6%22);)  [Edit](https://web-s-ebscohost-com.ezproxy.library.sydney.edu.au/Legacy/Views/UserControls/EHOST/) |
| S5 | (MH "Child, Preschool") | [View Results](javascript:__doPostBack('ctl00$ctl00$FindField$FindField$historyControl$HistoryRepeater$ctl32$linkResults','')) (223,269)  [View Details](javascript:showShDetails(%22ctl00_ctl00_FindField_FindField_historyControl_ctrlPopup%22,%20%22S5%22);)  [Edit](https://web-s-ebscohost-com.ezproxy.library.sydney.edu.au/Legacy/Views/UserControls/EHOST/) |
| S4 | (MH "Child+") | [View Results](javascript:__doPostBack('ctl00$ctl00$FindField$FindField$historyControl$HistoryRepeater$ctl33$linkResults','')) (722,453)  [View Details](javascript:showShDetails(%22ctl00_ctl00_FindField_FindField_historyControl_ctrlPopup%22,%20%22S4%22);)  [Edit](https://web-s-ebscohost-com.ezproxy.library.sydney.edu.au/Legacy/Views/UserControls/EHOST/) |
| S3 | TX infants or toddlers or early childhood | [View Results](javascript:__doPostBack('ctl00$ctl00$FindField$FindField$historyControl$HistoryRepeater$ctl34$linkResults','')) (454,627)  [View Details](javascript:showShDetails(%22ctl00_ctl00_FindField_FindField_historyControl_ctrlPopup%22,%20%22S3%22);)  [Edit](https://web-s-ebscohost-com.ezproxy.library.sydney.edu.au/Legacy/Views/UserControls/EHOST/) |
| S2 | TX infants or baby or newborn or neonate | [View Results](javascript:__doPostBack('ctl00$ctl00$FindField$FindField$historyControl$HistoryRepeater$ctl35$linkResults','')) (520,233)  [View Details](javascript:showShDetails(%22ctl00_ctl00_FindField_FindField_historyControl_ctrlPopup%22,%20%22S2%22);)  [Edit](https://web-s-ebscohost-com.ezproxy.library.sydney.edu.au/Legacy/Views/UserControls/EHOST/) |
| S1 | (MH "Infant+") | [View Results](javascript:__doPostBack('ctl00$ctl00$FindField$FindField$historyControl$HistoryRepeater$ctl36$linkResults','')) (279,328)  [View Details](javascript:showShDetails(%22ctl00_ctl00_FindField_FindField_historyControl_ctrlPopup%22,%20%22S1%22);)  [Edit](https://web-s-ebscohost-com.ezproxy.library.sydney.edu.au/Legacy/Views/UserControls/EHOST/) |

# Supplementary Table 4. Database search: Embase, dental caries.

| **#** | **Searches** | **Results** |
| --- | --- | --- |
| 1 | infant/ | 679650 |
| 2 | child/ or preschool child/ | 2171936 |
| 3 | 1 or 2 | 2464365 |
| 4 | dental caries/ | 54593 |
| 5 | "dental caries".mp. | 61219 |
| 6 | "tooth decay".mp. | 1948 |
| 7 | dental care.mp. or dental procedure/ | 87276 |
| 8 | "early childhood caries".mp. | 2069 |
| 9 | 4 or 5 or 6 or 7 or 8 | 142305 |
| 10 | attitude to health/ | 122264 |
| 11 | culture.mp. | 1543215 |
| 12 | health education/ | 103028 |
| 13 | health behavior/ | 73026 |
| 14 | knowledge/ or knowledge.mp. | 1055670 |
| 15 | social belief/ or health belief/ or belief.mp. | 61477 |
| 16 | perception.mp. or risk perception/ | 400140 |
| 17 | perspective.mp. | 304986 |
| 18 | 10 or 11 or 12 or 13 or 14 or 15 or 16 or 17 | 3415511 |
| 19 | parent/ or parent.mp. | 296370 |
| 20 | caregiver/ or caregiver.mp. | 115101 |
| 21 | carer.mp. | 8919 |
| 22 | guardian.mp. or legal guardian/ | 6304 |
| 23 | 19 or 20 or 21 or 22 | 408444 |
| 24 | 3 and 9 and 18 and 23 | 894 |
| 25 | qualitative.mp. or qualitative research/ | 390672 |
| 26 | unstructured interview/ or interview/ or structured interview/ or interview.mp. or semi structured interview/ | 393014 |
| 27 | "focus group".mp. | 41000 |
| 28 | 25 or 26 or 27 | 772105 |
| 29 | 24 and 28 | 156 |

# Supplementary Table 5. Database search: Medline, overweight/obesity.

| [# ▲](https://ovidsp-dc2-ovid-com.ezproxy.library.sydney.edu.au/ovid-b/ovidweb.cgi?&S=IAJDFPGKMBEBMCODIPMJKFLECCMDAA00&Sort+Sets=descending) | **Searches** | **Results** |
| --- | --- | --- |
| 1 | Infant/ | 842414 |
| 2 | Infant, Newborn/ | 645595 |
| 3 | Child/ | 1826940 |
| 4 | 1 or 2 or 3 | 2537197 |
| 5 | Obesity/ or Overweight/ | 213711 |
| 6 | "Weights and Measures"/ | 2677 |
| 7 | (body adj3 weight).mp. | 387257 |
| 8 | (body adj3 size).mp. | 39710 |
| 9 | 5 or 6 or 7 or 8 | 2380253 |
| 10 | Health Knowledge, Attitudes, Practice/ | 122875 |
| 11 | Culture/ | 33977 |
| 12 | Attitude to Health/ | 85338 |
| 13 | Health Behavior/ | 54678 |
| 14 | knowledge.mp. | 891835 |
| 15 | belief.mp. | 37826 |
| 16 | perception.mp. | 399844 |
| 17 | perspective.mp. | 253317 |
| 18 | 10 or 11 or 12 or 13 or 14 or 15 or 16 or 17 | 1631020 |
| 19 | Parents/ | 73805 |
| 20 | parent.mp. | 174471 |
| 21 | Caregivers/ | 44784 |
| 22 | caregiver.mp. | 34602 |
| 23 | carer.mp. | 5755 |
| 24 | guardian.mp. or Legal Guardians/ | 5412 |
| 25 | 19 or 20 or 21 or 22 or 23 or 24 | 284214 |
| 26 | Qualitative Research/ or qualitative.mp. | 299921 |
| 27 | Interview/ or interview.mp. | 177274 |
| 28 | "focus group".mp. or Focus Groups/ | 52044 |
| 29 | 26 or 27 or 28 | 500179 |
| 30 | 4 and 9 and 18 and 25 | 1032 |
| 31 | 29 and 30 | 155 |

# Supplementary Table 6. Database search: CINAHL, overweight/obesity.

| **Search Terms** | **Search Options** |  |
| --- | --- | --- |
| S37 | S30 AND S36 | [View Results](javascript:__doPostBack('ctl00$ctl00$FindField$FindField$historyControl$HistoryRepeater$ctl00$linkResults','')) (4,905)  [View Details](javascript:showShDetails(%22ctl00_ctl00_FindField_FindField_historyControl_ctrlPopup%22,%20%22S37%22);)  [Edit](https://web-s-ebscohost-com.ezproxy.library.sydney.edu.au/Legacy/Views/UserControls/Ehost/) |
| S36 | S31 OR S32 OR S33 OR S34 OR S35 | [View Results](javascript:__doPostBack('ctl00$ctl00$FindField$FindField$historyControl$HistoryRepeater$ctl01$linkResults','')) (615,408)  [View Details](javascript:showShDetails(%22ctl00_ctl00_FindField_FindField_historyControl_ctrlPopup%22,%20%22S36%22);)  [Edit](https://web-s-ebscohost-com.ezproxy.library.sydney.edu.au/Legacy/Views/UserControls/Ehost/) |
| S35 | TX "focus group" | [View Results](javascript:__doPostBack('ctl00$ctl00$FindField$FindField$historyControl$HistoryRepeater$ctl02$linkResults','')) (45,832)  [View Details](javascript:showShDetails(%22ctl00_ctl00_FindField_FindField_historyControl_ctrlPopup%22,%20%22S35%22);)  [Edit](https://web-s-ebscohost-com.ezproxy.library.sydney.edu.au/Legacy/Views/UserControls/Ehost/) |
| S34 | (MH "Focus Groups") | [View Results](javascript:__doPostBack('ctl00$ctl00$FindField$FindField$historyControl$HistoryRepeater$ctl03$linkResults','')) (47,931)  [View Details](javascript:showShDetails(%22ctl00_ctl00_FindField_FindField_historyControl_ctrlPopup%22,%20%22S34%22);)  [Edit](https://web-s-ebscohost-com.ezproxy.library.sydney.edu.au/Legacy/Views/UserControls/Ehost/) |
| S33 | (MH "Semi-Structured Interview") OR (MH "Structured Interview") | [View Results](javascript:__doPostBack('ctl00$ctl00$FindField$FindField$historyControl$HistoryRepeater$ctl04$linkResults','')) (82,327)  [View Details](javascript:showShDetails(%22ctl00_ctl00_FindField_FindField_historyControl_ctrlPopup%22,%20%22S33%22);)  [Edit](https://web-s-ebscohost-com.ezproxy.library.sydney.edu.au/Legacy/Views/UserControls/Ehost/) |
| S32 | TX qualitative research or qualitative study or qualitative methods or interview | [View Results](javascript:__doPostBack('ctl00$ctl00$FindField$FindField$historyControl$HistoryRepeater$ctl05$linkResults','')) (424,654)  [View Details](javascript:showShDetails(%22ctl00_ctl00_FindField_FindField_historyControl_ctrlPopup%22,%20%22S32%22);)  [Edit](https://web-s-ebscohost-com.ezproxy.library.sydney.edu.au/Legacy/Views/UserControls/Ehost/) |
| S31 | (MH "Qualitative Studies+") | [View Results](javascript:__doPostBack('ctl00$ctl00$FindField$FindField$historyControl$HistoryRepeater$ctl06$linkResults','')) (167,447)  [View Details](javascript:showShDetails(%22ctl00_ctl00_FindField_FindField_historyControl_ctrlPopup%22,%20%22S31%22);)  [Edit](https://web-s-ebscohost-com.ezproxy.library.sydney.edu.au/Legacy/Views/UserControls/Ehost/) |
| S30 | S6 AND S11 AND S23 AND S29 | [View Results](javascript:__doPostBack('ctl00$ctl00$FindField$FindField$historyControl$HistoryRepeater$ctl07$linkResults','')) (30,747)  [View Details](javascript:showShDetails(%22ctl00_ctl00_FindField_FindField_historyControl_ctrlPopup%22,%20%22S30%22);)  [Edit](https://web-s-ebscohost-com.ezproxy.library.sydney.edu.au/Legacy/Views/UserControls/Ehost/) |
| S29 | S24 OR S25 OR S26 OR S27 OR S28 | [View Results](javascript:__doPostBack('ctl00$ctl00$FindField$FindField$historyControl$HistoryRepeater$ctl08$linkResults','')) (1,246,631)  [View Details](javascript:showShDetails(%22ctl00_ctl00_FindField_FindField_historyControl_ctrlPopup%22,%20%22S29%22);)  [Edit](https://web-s-ebscohost-com.ezproxy.library.sydney.edu.au/Legacy/Views/UserControls/Ehost/) |
| S28 | TX ( parents or caregivers or mother or father or parent or family ) OR TX guardians OR TX carers | [View Results](javascript:__doPostBack('ctl00$ctl00$FindField$FindField$historyControl$HistoryRepeater$ctl09$linkResults','')) (1,245,281)  [View Details](javascript:showShDetails(%22ctl00_ctl00_FindField_FindField_historyControl_ctrlPopup%22,%20%22S28%22);)  [Edit](https://web-s-ebscohost-com.ezproxy.library.sydney.edu.au/Legacy/Views/UserControls/Ehost/) |
| S27 | (MH "Guardianship, Legal+") | [View Results](javascript:__doPostBack('ctl00$ctl00$FindField$FindField$historyControl$HistoryRepeater$ctl10$linkResults','')) (1,893)  [View Details](javascript:showShDetails(%22ctl00_ctl00_FindField_FindField_historyControl_ctrlPopup%22,%20%22S27%22);)  [Edit](https://web-s-ebscohost-com.ezproxy.library.sydney.edu.au/Legacy/Views/UserControls/Ehost/) |
| S26 | (MH "Caregivers") | [View Results](javascript:__doPostBack('ctl00$ctl00$FindField$FindField$historyControl$HistoryRepeater$ctl11$linkResults','')) (39,874)  [View Details](javascript:showShDetails(%22ctl00_ctl00_FindField_FindField_historyControl_ctrlPopup%22,%20%22S26%22);)  [Edit](https://web-s-ebscohost-com.ezproxy.library.sydney.edu.au/Legacy/Views/UserControls/Ehost/) |
| S25 | (MH "Foster Parents") | [View Results](javascript:__doPostBack('ctl00$ctl00$FindField$FindField$historyControl$HistoryRepeater$ctl12$linkResults','')) (1,054)  [View Details](javascript:showShDetails(%22ctl00_ctl00_FindField_FindField_historyControl_ctrlPopup%22,%20%22S25%22);)  [Edit](https://web-s-ebscohost-com.ezproxy.library.sydney.edu.au/Legacy/Views/UserControls/Ehost/) |
| S24 | (MH "Parents+") | [View Results](javascript:__doPostBack('ctl00$ctl00$FindField$FindField$historyControl$HistoryRepeater$ctl13$linkResults','')) (104,558)  [View Details](javascript:showShDetails(%22ctl00_ctl00_FindField_FindField_historyControl_ctrlPopup%22,%20%22S24%22);)  [Edit](https://web-s-ebscohost-com.ezproxy.library.sydney.edu.au/Legacy/Views/UserControls/Ehost/) |
| S23 | S12 OR S13 OR S14 OR S15 OR S16 OR S17 OR S18 OR S19 OR S20 OR S21 OR S22 | [View Results](javascript:__doPostBack('ctl00$ctl00$FindField$FindField$historyControl$HistoryRepeater$ctl14$linkResults','')) (1,907,993)  [View Details](javascript:showShDetails(%22ctl00_ctl00_FindField_FindField_historyControl_ctrlPopup%22,%20%22S23%22);)  [Edit](https://web-s-ebscohost-com.ezproxy.library.sydney.edu.au/Legacy/Views/UserControls/Ehost/) |
| S22 | TX perspective or perception or opinion or experience or attitude | [View Results](javascript:__doPostBack('ctl00$ctl00$FindField$FindField$historyControl$HistoryRepeater$ctl15$linkResults','')) (1,190,081)  [View Details](javascript:showShDetails(%22ctl00_ctl00_FindField_FindField_historyControl_ctrlPopup%22,%20%22S22%22);)  [Edit](https://web-s-ebscohost-com.ezproxy.library.sydney.edu.au/Legacy/Views/UserControls/Ehost/) |
| S21 | (MH "Health Behavior+") | [View Results](javascript:__doPostBack('ctl00$ctl00$FindField$FindField$historyControl$HistoryRepeater$ctl16$linkResults','')) (113,205)  [View Details](javascript:showShDetails(%22ctl00_ctl00_FindField_FindField_historyControl_ctrlPopup%22,%20%22S21%22);)  [Edit](https://web-s-ebscohost-com.ezproxy.library.sydney.edu.au/Legacy/Views/UserControls/Ehost/) |
| S20 | TX attitudes or perceptions or opinions or thoughts or feelings or beliefs | [View Results](javascript:__doPostBack('ctl00$ctl00$FindField$FindField$historyControl$HistoryRepeater$ctl17$linkResults','')) (753,256)  [View Details](javascript:showShDetails(%22ctl00_ctl00_FindField_FindField_historyControl_ctrlPopup%22,%20%22S20%22);)  [Edit](https://web-s-ebscohost-com.ezproxy.library.sydney.edu.au/Legacy/Views/UserControls/Ehost/) |
| S19 | (MH "Attitude to Health+") | [View Results](javascript:__doPostBack('ctl00$ctl00$FindField$FindField$historyControl$HistoryRepeater$ctl18$linkResults','')) (172,584)  [View Details](javascript:showShDetails(%22ctl00_ctl00_FindField_FindField_historyControl_ctrlPopup%22,%20%22S19%22);)  [Edit](https://web-s-ebscohost-com.ezproxy.library.sydney.edu.au/Legacy/Views/UserControls/Ehost/) |
| S18 | (MH "Health Education+") | [View Results](javascript:__doPostBack('ctl00$ctl00$FindField$FindField$historyControl$HistoryRepeater$ctl19$linkResults','')) (138,182)  [View Details](javascript:showShDetails(%22ctl00_ctl00_FindField_FindField_historyControl_ctrlPopup%22,%20%22S18%22);)  [Edit](https://web-s-ebscohost-com.ezproxy.library.sydney.edu.au/Legacy/Views/UserControls/Ehost/) |
| S17 | TX beliefs or perceptions or views or attitudes or opinions or knowledge | [View Results](javascript:__doPostBack('ctl00$ctl00$FindField$FindField$historyControl$HistoryRepeater$ctl20$linkResults','')) (923,585)  [View Details](javascript:showShDetails(%22ctl00_ctl00_FindField_FindField_historyControl_ctrlPopup%22,%20%22S17%22);)  [Edit](https://web-s-ebscohost-com.ezproxy.library.sydney.edu.au/Legacy/Views/UserControls/Ehost/) |
| S16 | (MH "Attitude to Illness+") | [View Results](javascript:__doPostBack('ctl00$ctl00$FindField$FindField$historyControl$HistoryRepeater$ctl21$linkResults','')) (24,605)  [View Details](javascript:showShDetails(%22ctl00_ctl00_FindField_FindField_historyControl_ctrlPopup%22,%20%22S16%22);)  [Edit](https://web-s-ebscohost-com.ezproxy.library.sydney.edu.au/Legacy/Views/UserControls/Ehost/) |
| S15 | (MH "Health Beliefs") | [View Results](javascript:__doPostBack('ctl00$ctl00$FindField$FindField$historyControl$HistoryRepeater$ctl22$linkResults','')) (14,827)  [View Details](javascript:showShDetails(%22ctl00_ctl00_FindField_FindField_historyControl_ctrlPopup%22,%20%22S15%22);)  [Edit](https://web-s-ebscohost-com.ezproxy.library.sydney.edu.au/Legacy/Views/UserControls/Ehost/) |
| S14 | (MH "Culture+") | [View Results](javascript:__doPostBack('ctl00$ctl00$FindField$FindField$historyControl$HistoryRepeater$ctl23$linkResults','')) (202,466)  [View Details](javascript:showShDetails(%22ctl00_ctl00_FindField_FindField_historyControl_ctrlPopup%22,%20%22S14%22);)  [Edit](https://web-s-ebscohost-com.ezproxy.library.sydney.edu.au/Legacy/Views/UserControls/Ehost/) |
| S13 | TX health knowledge or health information or health understanding | [View Results](javascript:__doPostBack('ctl00$ctl00$FindField$FindField$historyControl$HistoryRepeater$ctl24$linkResults','')) (166,731)  [View Details](javascript:showShDetails(%22ctl00_ctl00_FindField_FindField_historyControl_ctrlPopup%22,%20%22S13%22);)  [Edit](https://web-s-ebscohost-com.ezproxy.library.sydney.edu.au/Legacy/Views/UserControls/Ehost/) |
| S12 | TX health knowledge, attitudes, practice or knowledge, attitudes, practice | [View Results](javascript:__doPostBack('ctl00$ctl00$FindField$FindField$historyControl$HistoryRepeater$ctl25$linkResults','')) (5,168)  [View Details](javascript:showShDetails(%22ctl00_ctl00_FindField_FindField_historyControl_ctrlPopup%22,%20%22S12%22);)  [Edit](https://web-s-ebscohost-com.ezproxy.library.sydney.edu.au/Legacy/Views/UserControls/Ehost/) |
| S11 | S7 OR S8 OR S9 OR S10 | [View Results](javascript:__doPostBack('ctl00$ctl00$FindField$FindField$historyControl$HistoryRepeater$ctl26$linkResults','')) (447,328)  [View Details](javascript:showShDetails(%22ctl00_ctl00_FindField_FindField_historyControl_ctrlPopup%22,%20%22S11%22);)  [Edit](https://web-s-ebscohost-com.ezproxy.library.sydney.edu.au/Legacy/Views/UserControls/Ehost/) |
| S10 | TX body n3 weight | [View Results](javascript:__doPostBack('ctl00$ctl00$FindField$FindField$historyControl$HistoryRepeater$ctl27$linkResults','')) (155,912)  [View Details](javascript:showShDetails(%22ctl00_ctl00_FindField_FindField_historyControl_ctrlPopup%22,%20%22S10%22);)  [Edit](https://web-s-ebscohost-com.ezproxy.library.sydney.edu.au/Legacy/Views/UserControls/Ehost/) |
| S9 | TX body n3 size | [View Results](javascript:__doPostBack('ctl00$ctl00$FindField$FindField$historyControl$HistoryRepeater$ctl28$linkResults','')) (13,151)  [View Details](javascript:showShDetails(%22ctl00_ctl00_FindField_FindField_historyControl_ctrlPopup%22,%20%22S9%22);)  [Edit](https://web-s-ebscohost-com.ezproxy.library.sydney.edu.au/Legacy/Views/UserControls/Ehost/) |
| S8 | TX overweight or obesity or obese or fat or unhealthy weight or high bmi | [View Results](javascript:__doPostBack('ctl00$ctl00$FindField$FindField$historyControl$HistoryRepeater$ctl29$linkResults','')) (241,324)  [View Details](javascript:showShDetails(%22ctl00_ctl00_FindField_FindField_historyControl_ctrlPopup%22,%20%22S8%22);)  [Edit](https://web-s-ebscohost-com.ezproxy.library.sydney.edu.au/Legacy/Views/UserControls/Ehost/) |
| S7 | (MH "Obesity+") | [View Results](javascript:__doPostBack('ctl00$ctl00$FindField$FindField$historyControl$HistoryRepeater$ctl30$linkResults','')) (110,167)  [View Details](javascript:showShDetails(%22ctl00_ctl00_FindField_FindField_historyControl_ctrlPopup%22,%20%22S7%22);)  [Edit](https://web-s-ebscohost-com.ezproxy.library.sydney.edu.au/Legacy/Views/UserControls/Ehost/) |
| S6 | S1 OR S2 OR S3 OR S4 OR S5 | [View Results](javascript:__doPostBack('ctl00$ctl00$FindField$FindField$historyControl$HistoryRepeater$ctl31$linkResults','')) (878,786)  [View Details](javascript:showShDetails(%22ctl00_ctl00_FindField_FindField_historyControl_ctrlPopup%22,%20%22S6%22);)  [Edit](https://web-s-ebscohost-com.ezproxy.library.sydney.edu.au/Legacy/Views/UserControls/Ehost/) |
| S5 | (MH "Child, Preschool") | [View Results](javascript:__doPostBack('ctl00$ctl00$FindField$FindField$historyControl$HistoryRepeater$ctl32$linkResults','')) (224,549)  [View Details](javascript:showShDetails(%22ctl00_ctl00_FindField_FindField_historyControl_ctrlPopup%22,%20%22S5%22);)  [Edit](https://web-s-ebscohost-com.ezproxy.library.sydney.edu.au/Legacy/Views/UserControls/Ehost/) |
| S4 | (MH "Child+") | [View Results](javascript:__doPostBack('ctl00$ctl00$FindField$FindField$historyControl$HistoryRepeater$ctl33$linkResults','')) (727,870)  [View Details](javascript:showShDetails(%22ctl00_ctl00_FindField_FindField_historyControl_ctrlPopup%22,%20%22S4%22);)  [Edit](https://web-s-ebscohost-com.ezproxy.library.sydney.edu.au/Legacy/Views/UserControls/Ehost/) |
| S3 | TX infants or toddlers or early childhood | [View Results](javascript:__doPostBack('ctl00$ctl00$FindField$FindField$historyControl$HistoryRepeater$ctl34$linkResults','')) (438,073)  [View Details](javascript:showShDetails(%22ctl00_ctl00_FindField_FindField_historyControl_ctrlPopup%22,%20%22S3%22);)  [Edit](https://web-s-ebscohost-com.ezproxy.library.sydney.edu.au/Legacy/Views/UserControls/Ehost/) |
| S2 | TX infants or baby or newborn or neonate | [View Results](javascript:__doPostBack('ctl00$ctl00$FindField$FindField$historyControl$HistoryRepeater$ctl35$linkResults','')) (451,900)  [View Details](javascript:showShDetails(%22ctl00_ctl00_FindField_FindField_historyControl_ctrlPopup%22,%20%22S2%22);)  [Edit](https://web-s-ebscohost-com.ezproxy.library.sydney.edu.au/Legacy/Views/UserControls/Ehost/) |
| S1 | (MH "Infant+") | [View Results](javascript:__doPostBack('ctl00$ctl00$FindField$FindField$historyControl$HistoryRepeater$ctl36$linkResults','')) (281,160)  [View Details](javascript:showShDetails(%22ctl00_ctl00_FindField_FindField_historyControl_ctrlPopup%22,%20%22S1%22);)  [Edit](https://web-s-ebscohost-com.ezproxy.library.sydney.edu.au/Legacy/Views/UserControls/Ehost/) |

# Supplementary Table 7. Database search: Embase, overweight/obesity.

| **[# ▲](https://ovidsp-dc2-ovid-com.ezproxy.library.sydney.edu.au/ovid-a/ovidweb.cgi?&S=BPMNFPHMBCEBKLAIJPNJJFDGKJDOAA00&Sort+Sets=descending)** | **Searches** | **Results** |
| --- | --- | --- |
| 1 |  infant/ | 685889 |
| 2 |  baby/ | 23738 |
| 3 |  child/ or preschool child/ | 2194174 |
| 4 |  1 or 2 or 3 | 2502409 |
| 5 |  obesity/ | 485849 |
| 6 |  overweight.mp. | 122261 |
| 7 |  (body adj3 size).mp. | 44979 |
| 8 |  (body adj3 weight).mp. | 663925 |
| 9 |  5 or 6 or 7 or 8 | 1136165 |
| 10 |  attitude to health/ | 123196 |
| 11 |  culture.mp. | 1556870 |
| 12 |  health education/ | 103902 |
| 13 |  health behavior/ | 73814 |
| 14 |  knowledge/ or knowledge.mp. | 1071042 |
| 15 |  social belief/ or health belief/ or belief.mp. | 62262 |
| 16 |  perception.mp. or risk perception/ | 405256 |
| 17 |  perspective.mp. | 310085 |
| 18 |  10 or 11 or 12 or 13 or 14 or 15 or 16 or 17 | 3454252 |
| 19 |  parent/ or parent.mp. | 298978 |
| 20 |  caregiver/ or caregiver.mp. | 117246 |
| 21 |  carer.mp. | 9033 |
| 22 |  guardian.mp. or legal guardian/ | 6424 |
| 23 |  19 or 20 or 21 or 22 | 413109 |
| 24 |  4 and 9 and 18 and 23 | 2077 |
| 25 |  qualitative.mp. or qualitative research/ | 397763 |
| 26 |  unstructured interview/ or interview/ or structured interview/ or interview.mp. or semi structured interview/ | 398151 |
| 27 |  "focus group".mp. | 41000 |
| 28 |  25 or 26 or 27 | 772105 |
| 32 |  27 and 31 | 403 |
