# Supplementary material for: Carer perspectives on overweight, obesity and dental caries in early childhood: findings from a systematic qualitative review
Source: Front Oral Health. 2025 Jun 18;6:1524715. doi: 10.3389/froh.2025.1524715 (PMC12213562; doi:10.3389/froh.2025.1524715)
Supplement: Supplementary file 3 [file Table3.docx]

Supplementary File 8

# Supplementary Table 8. Reflexivity in coding for thematic analysis.

| **Text** | **Coding** |
| --- | --- |
| *“Well, I think it doesn’t matter if babies are chubby or overweight, because that’s something they will outgrow. When they start to move around you often see the baby fat disappear. So I worry less about a chubby baby.”*  Bektas, G., F. Boelsma, V. E. Baur, J. C. Seidell and S. C. Dijkstra (2020). "Parental Perspectives and Experiences in Relation to Lifestyle-Related Practices in the First Two Years of a Child’s Life: A Qualitative Study in a Disadvantaged Neighborhood in The Netherlands." International Journal of Environmental Research and Public Health 17(16): 5838. <https://doi.org/10.3390/ijerph17165838> | - Positive sentiment   - Positive perspective towards ‘chubby or overweight’ babies - Beliefs or knowledge 🡪 infant or child weight 🡪 largeness or heaviness - Motivation 🡪 response or reaction to infant/child overweight/obesity   - Perspective towards large or heavy infant, described as ‘chubby or overweight’ - Beliefs or knowledge 🡪 infant or child weight 🡪 health not dependent on weight   - Perspective that ‘it doesn’t matter if babies are chubby or overweight’ - Beliefs or knowledge 🡪 infant or child weight 🡪 short-term, temporary, non-permanent condition   - Belief that being at a higher body weight is ‘something they will outgrow’ - Beliefs or knowledge 🡪 physical activity or play   - Belief that a higher body weight is resolved when infant mobility increases - Motivation 🡪 values 🡪 parenting style or approach   - Perspective towards concerns about large or heavy infant |
| *Many parents also believed that their infants should be familiar with using screens from an early age, so that they can develop their digital skills early on. Parents also reported that they considered television, tablet or smartphone viewing to be educational and an important element of their infant’s visual, cognitive, and social development. A considerable proportion of respondents said their infant was allowed about 30–60 min of screen time per day for educational purposes.*  *“I give him the iPad when I sleep, so that he can watch cartoons in Arabic. I want him to learn both Arabic and Dutch.”*  Bektas, G., F. Boelsma, V. E. Baur, J. C. Seidell and S. C. Dijkstra (2020). "Parental Perspectives and Experiences in Relation to Lifestyle-Related Practices in the First Two Years of a Child’s Life: A Qualitative Study in a Disadvantaged Neighborhood in The Netherlands." International Journal of Environmental Research and Public Health 17(16): 5838. <https://doi.org/10.3390/ijerph17165838> | Coded with:   - Positive sentiment   - Positive perspective towards use of devices and screens - Motivation 🡪 values 🡪 parenting style or approach - Beliefs or knowledge 🡪 sedentary behaviour - Beliefs or knowledge 🡪 other   - Perspectives towards value of devices and screens for infant development |
| *Parents commonly described adding sugar to cereal or water for children to “make it taste better.” Sugar cravings were discussed in terms of parent addiction as well as children; children would often cry or throw a tantrum until parents succumbed to the child’s demands.*  *Some parents justified giving sugar because of their cravings: “You can’t expect them to give up because I’m addicted to Coke, you know, so, just give it.”*  *Using sugar to spoil children was common, even for children who were not yet on solids: “I only give her honey on the dummy every now and then because I like just to give her treats but it’s not all the time.”*  Poirier, B., J. Hedges, L. Smithers, M. Moskos and L. Jamieson (2021). ""What are we doing to our babies' teeth?" Barriers to establishing oral health practices for Indigenous children in South Australia." BMC Oral Health 21(1): 1-12. <https://doi.org/10.1186/s12903-021-01791-x> | - Positive sentiment   - Positive perspective towards palatability of sugar-sweetened foods and provision of treats for children - Motivation 🡪 values 🡪 parenting style or approach - Motivation 🡪 values 🡪 child feeding - Capability 🡪 self-efficacy - Beliefs or knowledge 🡪 food or feeding 🡪 foods   - Perspective towards use of food to settle child behaviour   - Beliefs towards role of sugar in child feeding (settling, treats) - Motivation 🡪 values 🡪 parenting style or approach - Motivation 🡪 response or reaction to infant or child cues - Motivation 🡪 values 🡪 child feeding - Beliefs or knowledge 🡪 food or feeding 🡪 foods   - Perspective towards value of food to demonstrate parental care   - Beliefs towards role of honey in child feeding (treats) - Opportunity 🡪 ability to change - Capability 🡪 self-efficacy   - Perspective towards ability to minimise children’s exposure to sugar   Perspective towards ability to settle child’s behaviour |
| *One brief story revealed the deep divide between Māori traditional beliefs about children and child-rearing, and the contemporary pressures that are undermining Māori traditions. A participant who worked at a Kōhanga Reo (a Māori preschool language and education center) spoke about the compassion they felt for a child who had been put on a diet. They disagreed with what they perceived as punitive, restrictive, and isolating treatment of the child, which led them to replace the child’s carrot stick lunch with what the other children were eating.*  *“We had one girl in the Kōhanga I was working in, and because her parents were overweight, obese. Overweight they were. They put their child on a diet and that was torture for us to see the rest of the kids eating yummy kai, and she’s got like carrot sticks and, you know, all this other healthy stuff…*  *So, we’d just like put her lunch away and give her a cake! [laughing] Because that is torture, it really is. You’re punishing the child because of your actions, you know? Because you’re overweight, you’re scared your child’s gonna get overweight, so you put them on a diet. Like, it’s a child. It’s torture for them.”*  Glover, M., Wong, S. F., Taylor, R. W., Derraik, J. G. B., Fa'alili-Fidow, J., Morton, S. M., & Cutfield, W. S. (2019). The Complexity of Food Provisioning Decisions by Māori Caregivers to Ensure the Happiness and Health of Their Children. Nutrients 11(5): 994. <https://doi.org/10.3390/nu11050994> | - Motivation 🡪 values 🡪 parenting style or approach - Motivation 🡪 values 🡪 child feeding - Beliefs or knowledge 🡪 infant or child weight 🡪 largeness or heaviness - Beliefs or knowledge 🡪 infant or child weight 🡪 response or reaction to overweight or obesity   - Perspective towards prioritising enjoyable eating experiences   - Response to feeding decisions made because of child’s reported weight status - Motivation 🡪 values 🡪 parenting style or approach - Motivation 🡪 response or reaction to infant or child cues - Motivation 🡪 values 🡪 child feeding   - Perspective towards value of food to demonstrate care   - Perspective towards prioritising enjoyable eating experiences |
| *Having satiated, and thus happy, children was what was most important to parents and caregivers in these focus groups. Weight was not perceived to be an appropriate focus for research, especially when some children were perceived to be under-nourished.*  *…*  *The most important focus in terms of child wellbeing was that children were not sick, and that they were developing physically, cognitively, and emotionally as well as could be expected. A healthy child was happy, active, and, as described by one South Auckland grandmother, “nice and open and they’re not scared... And they speak their mind.” The parents advised not intervening when there was not a problem.*  Glover, M., Wong, S. F., Taylor, R. W., Derraik, J. G. B., Fa'alili-Fidow, J., Morton, S. M., & Cutfield, W. S. (2019). The Complexity of Food Provisioning Decisions by Māori Caregivers to Ensure the Happiness and Health of Their Children. Nutrients 11(5): 994. <https://doi.org/10.3390/nu11050994> | - Positive sentiment   - Positive perspective towards happy and adequately fed children   - Positive perspective towards child well-being and priorities for child health - Negative sentiment   - Negative perspective towards focus on child weight - Motivation 🡪 values 🡪 parenting style or approach - Opportunity 🡪 cultural beliefs and norms - Beliefs or knowledge 🡪 infant or child weight 🡪 health not dependent on weight - Beliefs or knowledge 🡪 mental health or self-esteem   - Cultural beliefs and norms in child well-being   - Perspective and priorities towards definitions of child well-being   - Perspective and priorities towards physical, cognitive and emotional wellness and child weight status |

Text cited from open-access journal articles.
