# Supplementary material for: Carer perspectives on overweight, obesity and dental caries in early childhood: findings from a systematic qualitative review
Source: Front Oral Health. 2025 Jun 18;6:1524715. doi: 10.3389/froh.2025.1524715 (PMC12213562; doi:10.3389/froh.2025.1524715)
Supplement: Supplementary file 4 [file Table4.docx]

Supplementary File 9

# Supplementary Table 9. Inter-rater reliability assessment CASP Qualitative Checklist quality appraisal.

| **Assessor** | **Reference** | **Clear statement of research aims** | **Appropriate qualitative method** | **Appropriate research design** | **Appropriate recruitment strategy** | **Data collection addressed research issue** | **Researcher-participant relationship** | **Ethical issues** | **Rigorous data analysis** | **Clear statement of findings** | **Results will help locally** |
| --- | --- | --- | --- | --- | --- | --- | --- | --- | --- | --- | --- |
| Reviewer A | Athavale 2020 | Yes | Yes | Yes | Unclear | Unclear | No | Yes | No | Unclear | Yes |
| Reviewer B | Athavale 2020 | Yes | Yes | Yes | Unclear | Unclear | No | Yes | No | Unclear | No |
| Reviewer A | Bentley 2017 | Yes | Yes | Yes | Yes | Yes | No | Yes | Unclear | Unclear | Yes |
| Reviewer B | Bentley 2017 | Yes | Yes | Yes | Yes | Yes | No | Yes | Unclear | Yes | Yes |
| Reviewer A | Finlayson 2019 | Yes | Yes | Yes | Yes | Yes | Unclear | Yes | Yes | Yes | Yes |
| Reviewer B | Finlayson 2019 | Yes | Yes | Yes | Yes | Yes | Unclear | Unclear | Yes | Yes | Yes |
| Reviewer A | Guendelman 2010 | Yes | Yes | Yes | Yes | Yes | No | No | No | Yes | Yes |
| Reviewer B | Guendelman 2010 | Yes | Yes | Yes | Yes | Yes | No | No | No | Yes | Yes |
| Reviewer A | Hardy 2019 | Yes | Yes | Yes | Yes | Yes | Yes | Yes | Yes | Yes | Yes |
| Reviewer B | Hardy 2019 | Yes | Yes | Yes | Yes | Yes | Yes | Yes | Yes | Yes | Yes |
| Reviewer A | Isong 2012 | Yes | Yes | Yes | Yes | Yes | Unclear | Yes | Yes | Yes | Yes |
| Reviewer B | Isong 2012 | Yes | Yes | Yes | Yes | Yes | Unclear | Yes | Yes | Yes | Yes |
| Reviewer A | Momeni 2017 | Yes | Yes | Yes | Yes | Yes | Unclear | Yes | No | Unclear | Yes |
| Reviewer B | Momeni 2017 | Yes | Yes | Yes | Yes | Yes | Unclear | Yes | No | Yes | Yes |
| Reviewer A | Nicol 2014 | Yes | Yes | Yes | Yes | Yes | Yes | Yes | Yes | Yes | Yes |
| Reviewer B | Nicol 2014 | Yes | Yes | Yes | Yes | Yes | Yes | Yes | Yes | Yes | Yes |
| Reviewer A | Patino-Fernandez 2013 | Yes | Yes | Yes | Yes | Yes | No | Unclear | Unclear | Yes | Yes |
| Reviewer B | Patino-Fernandez 2013 | Yes | Yes | Yes | Yes | Yes | No | Unclear | Yes | Yes | Unclear |
| Reviewer A | Poirer 2021 | Yes | Yes | Yes | Yes | Unclear | Yes | Yes | Yes | Yes | Yes |
| Reviewer B | Poirer 2021 | Yes | Yes | Yes | Yes | Yes | Yes | Yes | Yes | Yes | Yes |
| Percentage agreement | 93.6 (overall) | 100 | 100 | 100 | 100 | 90.9 | 100 | 90.9 | 90.9 | 81.8 | 81.8 |
