# Supplementary material for: Carer perspectives on overweight, obesity and dental caries in early childhood: findings from a systematic qualitative review
Source: Front Oral Health. 2025 Jun 18;6:1524715. doi: 10.3389/froh.2025.1524715 (PMC12213562; doi:10.3389/froh.2025.1524715)
Supplement: Supplementary file 5 [file Table5.docx]

Supplementary File 10

# Supplementary Table 10. Descriptive characteristics of included articles (N=98).

| **First author, year** | **Country** | **Study period** | **Participants – adults** | **Infant/child age** | **Participants not coded** | **Diagnosed with ECC or O/O? (prevalence in children)** | **Increased risk of ECC or O/O? + risk factors** | **Qualitative research method** |
| --- | --- | --- | --- | --- | --- | --- | --- | --- |
| Amin 2012 | Canada | NR | 48 mothers | 3-5 yrs |  |  | Immigrants, refugees | Focus group |
| Amin 2009 | Canada | NR | 14 mothers, 5 fathers | 2.5-6 yrs |  | ECC (18/18) |  | SSI |
| Amin 2006 | Canada | NR | 8 mothers, 3 fathers | 2.5-6 yrs |  | ECC (11/11) |  | SSI |
| Arora 2021 | Australia | Oct 2017-Dec 2018 | 45 mothers | 2-3 yrs |  | ECC (9/38) | Disadvantaged area | SSI |
| Athavale 2020 | India | 2012, 2015 | 23 mothers or carers, 14 pregnant women | 6 mo - 6 yrs | Health workers |  | Low income | Focus group |
| Balasooriyan 2024 | The Netherlands | Mar 2022-Mar 2023 | 47 mothers, 5 fathers | <4 yrs |  | ECC (qualitatively reported) | Disadvantaged area | SSI, focus group, observation |
| Baughcum 1998 | USA | Aug-Oct 1997 | 14 mothers | 1-3 yrs |  |  | Low income, cultural minority, adolescent mothers | Focus group |
| Beck 2018 | USA | Oct 2013-May 2014 | 25 mothers, 3 fathers | 4 mo - 2 yrs |  |  | Low income, cultural minority | SSI |
| Bektas 2020 | The Netherlands | NR | 38 parents | 0-2 yrs |  |  | Immigrants, cultural minority, disadvantaged area | Focus group, SSI |
| Bentley 2017 | UK | Jun-Aug 2014 | 19 mothers, 1 father | <12 mo |  |  | High obesity area | SSI |
| Brotanek 2009 | USA | NR | 39 parents | 15-48 mo |  |  | Immigrants, cultural minority | SSI |
| Butten 2020 | Australia | Aug 2017 | 19 mothers, 1 aunt | <5 yrs |  |  | Indigenous population | Yarning (focus group) |
| Cave 2021 | New Zealand | Jun-Sept 2019 | 16 mothers, 3 female carers | 4-5 yrs |  | Obesity (19/19) | Indigenous population | Focus group, SSI |
| Cespedes 2012 | Mexico | Jul 2010-Jun 2011 | 43 mothers, 9 aunts, grandmothers or fathers | 2-5 yrs |  | O/O (52/52) | Low income, cultural minority | Interview |
| Chaidez 2011 | USA | NR | 18 mothers | 12-47 mo |  |  | Cultural minority | Interview |
| Cheney 2019 | USA | Sept 2015-May 2016 | 19 mothers | <2 yrs |  |  | Low income, cultural minority | Focus group |
| Collins 2016 | USA | Aug 2015 | 8 mothers, 2 fathers | 6 mo - 6 yrs |  |  | Low income | Photovoice |
| Crawford 2004 | USA | NR | 43 mothers | 2-5 yrs |  |  | Low income, cultural minority | Focus group |
| Custodio 2019 | Brazil | Jan-Jul 2017 | 10 mothers | 3-8 yrs; <6 yrs coded | Mothers of 7-8 yrs children | ECC (10/10) |  | SSI |
| Dinkel 2017 | USA | May 2015-Mar 2016 | 11 mothers | ~6 mo |  | Overweight (11/29) |  | SSI |
| Do 2016 | Vietnam | Jan-Feb 2015 | 33 mothers | 4-6 yrs |  | O/O (16/33) | Country in economic transition | Focus group |
| Ek 2020 | Sweden | NR | 24 mothers, 12 fathers | 4-6 yrs |  | Obesity (35/35) |  | SSI |
| Eli 2014 | USA | Feb-May 2011 | 22 parents, 27 grandparents | 3-5 yrs |  | O/O (9/16) | Low income, low education | SSI |
| Elwell 2021 | USA | Feb-May 2019 | 20 mothers or pregnant women, 7 carers | <6 yrs | Health workers | ECC (qualitatively reported) | Indigenous population | SSI |
| Finlayson 2019 (Hispanic mothers) | USA | Aug 2010 | 36 mothers | 3-5 yrs |  |  | Low income, cultural minority | Focus group, interview |
| Finlayson 2019 (Early Head Start) | USA | Nov 2016-Feb 2017 | 25 mothers | 6 mo - 4 yrs |  | ECC (qualitatively reported) | Low income | SSI |
| Gainsbury 2018 | UK | NR | 15 mothers, 3 fathers | 4-5 yrs |  | O/O (8/18) | High obesity area | Focus group |
| Galvez 2022 | Chile | NR | 25 parents | 3-5 yrs |  |  | Low income | SSI |
| Glover 2019 | New Zealand | NR | 39 parents, 2 grandmothers, 2 aunts | 6 mo - 5 yrs |  |  | Indigenous population | Focus group |
| Goodell 2008 | USA | NR | 73 parents | 2-5 yrs |  | Overweight (qualitatively reported) | Low income, cultural minority | Focus group, interview |
| Guendelman 2010 | USA | Mar 2006-Jan 2008 | 84 mothers | 4-6 yrs |  |  | Low income, cultural minority | Focus group |
| Guerrero 2011 | USA | NR | 24 mothers | 2-5 yrs |  | Overweight (12/24) | Low income, cultural minority | Focus group |
| Hardy 2019 | Australia | Jun 2017 | 10 mothers | 2-5 yrs |  | O/O (10/10) |  | SSI |
| Hashim 2010 | United Arab Emirates | Nov 2003-May 2004 | 32 mothers | 5 yrs |  |  | Low education, country in economic transition | Focus group |
| Hilton 2007 | USA | NR | 177 carers | 1-5 yrs |  | ECC (qualitatively reported) | Cultural minority | Focus group |
| Hoeft 2010 | USA | NR | 48 mothers | <5 yrs |  | ECC (60% of <5 yrs children) | Low income, cultural minority | SSI |
| Hoeft 2015 | USA | NR | 45 mothers, 6 grandmothers or aunts or foster parents | 1-5 yrs |  | ECC (qualitatively reported) | Low income, cultural minority | Focus group |
| Horowitz 2016 | USA | July 2011 | 16 mothers or pregnant women | <6 yrs |  |  | Low income, low education, cultural minority | Focus group |
| Horowitz 2015 | USA | 2010 | 22 mothers, 2 fathers, 1 grandfather, 8 pregnant women | <6 yrs |  |  | Low income | Focus group |
| Horton 2009 | USA | Sept 2005-May 2006 | 26 mothers | <6 yrs |  | ECC (qualitatively reported) | Low income, immigrants, cultural minority | Interview, observations |
| Horton 2008 | USA | Sept 2005-May 2006 | 38 mothers | <6 yrs |  | ECC (23/38) | Low income, immigrants, cultural minority | Interview, observations |
| Hughes 2010 | USA | Jan-Feb 2000 | 21 mothers | 30-60 mo |  | O/O (21/21) | Low income | SSI |
| Isong 2012 | USA | Jun-Dec 2009 | 25 parents | 2-5 yrs |  | ECC (25/25) |  | SSI |
| Jain 2001 | USA | Nov-Dec 1999 | 18 mothers | 2-5 yrs |  | O/O (17/18) | Low income | Focus group |
| Karasz 2014 | USA | NR | 13 mothers | 6 mo - 2 yrs | Pediatricians | ECC (qualitatively reported) | Immigrants, cultural minority | Focus group, interview |
| Keenan 2010 | UK | NR | 60 mothers | <2 yrs |  | O/O (qualitatively reported) | Mother with obesity | Interview |
| Klingberg 2020 | South Africa | Sept 2018-Jan 2019 | 13 mothers, 2 father, 1 aunt | 3-5 yrs |  |  | Low income, mixed employment | SSI |
| Lakhanpaul 2020 | UK | NR | 21 mothers, 10 fathers, 28 grandmothers, 11 grandfathers | 6-23 mo | Health professionals, key informants, community members |  | Low income area, low employment area, cultural minority area | Focus group, SSI |
| Lee 2022 | Canada | Oct-Dec 2019 | 6 parents, 1 grandparent | <6 yrs |  | ECC (qualitatively reported) | Indigenous population | Digital story video, SSI |
| Lindsay 2017 | USA | Mar-May 2015 | 29 mothers | 2-5 yrs |  |  | Immigrants, cultural minority | Focus group |
| Lindsay 2012 | Argentina | Dec 2006 | 38 mothers | 2-5 yrs |  | Overweight (qualitatively reported) | Low income | Focus group |
| Lindsay 2009 | Brazil | Jun-Aug 2004 | 41 mothers | 1-3 yrs |  | Overweight (qualitatively reported) | Low income | Focus group |
| Lindsay 2011 | USA | Sept 2005-Feb 2006 | 51 mothers | <4 yrs |  |  | Low income, cultural minority | Focus group, interview |
| López del Valle 2005 | Puerto Rico | NR | 150 mothers or grandmothers or pregnant women | <5 yrs |  |  | Low income | Focus group |
| Masterson 2014 | USA | Jul 2006-Aug 2007 | 37 mothers | 5 yrs |  | ECC (qualitatively reported) | Low income, immigrants, cultural minority | SSI |
| McDonald 2015 | USA | Oct 2011-Jan 2012 | 12 mothers | 2-5 yrs |  | O/O (12/12) | Cultural minority | SSI |
| McFarren 2020 | USA | Dec 2018-Feb 2019 | 14 parents | 6-18 mo |  | Obesity (14/14) | Cultural minority | SSI |
| McGarvey 2006 | USA | NR | 24 mothers, 1 father | <5 yrs |  | Overweight (qualitatively reported) | Low income, cultural minority | Focus group |
| Mofidi 2009 | USA | Oct-Dec 2004 | 21 mothers, 1 father, 13 pregnant women | 3-5 yrs | Early childhood care staff | ECC (qualitatively reported) | Low income, cultural minority | Focus group |
| Momeni 2019 | Iran | Apr-May 2015 | 58 mothers | 6 yrs |  |  | Mixed education, country in economic transition | Focus group, SSI |
| Momeni 2017 | Iran | Apr-May 2015 | 58 mothers | 6 yrs |  |  | Mixed education, country in economic transition | Focus group, SSI |
| Naidu 2012 | Trinidad and Tobago | Nov 2010-Feb 2011 | 18 carers | 3-5 yrs |  |  | Mixed ethnicity, under-researched country | Focus group |
| Nicely 2019 | USA | Oct 2016-Feb 2017 | 24 mothers, 1 father, 3 grandparents | 3-5 yrs |  | O/O (qualitatively reported) | Low income | Focus group |
| Nicol 2014 | Australia | Dec 2012-Feb 2013 | 39 parents, carers or grandparents | <5 yrs | Nurses | ECC (qualitatively reported) | Refugees, cultural minority | Focus group |
| Patino-Fernandez 2013 | USA | Nov 2007-Jan 2008 | 8 mothers, 1 father | 6 yrs |  | Overweight (qualitatively reported) | Low income, cultural minority | Focus group |
| Poirier 2021 (barriers to oral health) | Australia | NR | 226 parents | 6mo - 6 yrs |  |  | Indigenous population | Interview |
| Poirier 2021 (motivations for oral health) | Australia | NR | 227 parents | 6mo - 6 yrs |  |  | Indigenous population | Interview |
| Poirier 2022 | Australia | Feb 2009-May 2013 | 226 parents | 6mo - 6 yrs |  |  | Indigenous population | Interview |
| Porter 2016 | USA | NR | 32 mothers | 3-5 yrs |  | O/O (8/23) | Low income, cultural minority | Focus group |
| Prowse 2014 | Canada | NR | 40 carers | <6 yrs |  | ECC (qualitatively reported) | Low income, immigrants, refugees, cultural minority, Indigenous population | Focus group |
| Rachmi 2017 | Indonesia | Jun-Oct 2016 | 46 mothers or grandmothers | <5 yrs | Carers of children aged 7-12 yrs or no specified age | O/O (qualitatively reported) | Low income, country in economic transition | Focus group |
| Redsell 2010 | UK | NR | 36 mothers, 2 fathers | <12 mo | Parents from area with low rates of childhood obesity |  | Low income, high obesity area | Focus group |
| Rich 2005 | USA | NR | 74 mothers, 1 father, 1 grandmother | <5 yrs |  | O/O (74/74) | Low income, cultural minority | Interview |
| Riggs 2015 | Australia | NR | 115 mothers, grandmothers, carers total | <12 yrs; <6 yrs coded | Carers of children aged 7-12 yrs or no specified age |  | Immigrants, refugees, cultural minority | Focus group |
| Rivera 2020 | USA | NR | 12 mothers, 6 fathers, 2 grandmothers | <5 yrs |  | ECC (4/20) | Low income, cultural minority, migrants | SSI |
| Rodríguez-Oliveros 2011 | Mexico | 2010 | 38 parents | ~5 yrs |  |  | Country in economic transition | Focus group |
| Roguski 2020 | New Zealand | NR | 107 parents or carers | 4 mo-6 yrs |  | ECC (qualitatively reported) | Indigenous population, low income | Focus group |
| Safaiyan 2021 | Iran | 2018-2019 | 37 mothers | 5-6 yrs |  | O/O (~12/37) | Country in economic transition, ≥50^th^ BMI percentile | SSI |
| Shrikrishna Suprabha 2022 | India | Mar-Aug 2019 | 22 mothers, 5 fathers | <6 yrs |  | ECC (27/27) |  | Focus group |
| Small 2009 | USA | NR | 9 mothers, 2 fathers | 3-6 yrs |  |  | Immigrants, cultural minority | Focus group |
| Steinman 2010 | USA | Sept-Nov 2006 | 37 mothers | <19 mo |  |  | Low income, cultural minority | Focus group |
| Suprabha 2021 | India | Mar-Aug 2019 | 22 mothers, 5 fathers | <6 yrs |  | ECC (27/27) |  | Focus group |
| Suprabha 2022 | India | Mar-Aug 2019 | 22 mothers, 5 fathers | <6 yrs |  | ECC (27/27) |  | Focus group |
| Suprabha 2024 | India | Mar-Aug 2019 | 22 mothers, 5 fathers | <6 yrs |  | ECC (27/27) |  | Focus group |
| Suprawoto 2019 | Indonesia | NR | 10 mothers | 2-6 yrs |  | Overweight (10/10) | Country in economic transition | Interview |
| Sussner 2008 | USA | Sept 2005-Feb 2006 | 51 mothers | <4 yrs |  |  | Low income, immigrants, cultural minority | Focus group, interview |
| Syrad 2015 | UK | NR | 26 parents | 4-5 yrs | Parents of children aged 10-11 yrs | O/O (10/10) | Mixed income, mixed ethnicity | SSI |
| Thompson 2014 | USA | NR | 20 mothers | <2 yrs |  | Overweight (qualitatively reported) | Low income, cultural minority | SSI |
| Tiwari 2017 | USA | NR | 20 mothers, 7 grandmothers, 1 father, 2 aunts | <6 yrs |  | ECC (qualitatively reported) | Low income, cultural minority | Focus group |
| Tiwari 2021 | USA | Jul 2017-Mar 2019 | 49 parents | <6 yrs |  | ECC (22/49) | Cultural minority | SSI |
| Toftemo 2013 | Norway | Mar-Sept 2012 | 10 mothers, 1 father | 2.5-5 yrs |  | Overweight (10/10) |  | SSI |
| Valencia 2016 | USA | Mar-Oct 2013 | 34 mothers, 19 carers | <6 mo |  |  | Low income, cultural minority | Focus group |
| van Nes 2018 | The Netherlands | NR | 29 mothers | <5 yrs |  | ECC (qualitatively reported) | Cultural minority | Focus group, SSI |
| Virgo-Milton 2016 | Australia | Mar-Jun 2011 | 32 mothers | 4-12 mo |  |  | Low income area, low employment area, cultural minority area | SSI |
| Weinstein 1999 | USA | NR | 62 pregnant women or parents/carers | <3 yrs |  |  | Low income, Indigenous population | Interview |
| Woolford 2007 | USA | Jun-Dec 2005 | 20 mothers | ~4 ys |  | O/O (4/20) | Low income | SSI |
| Wu 2021 | China | Oct-Dec 2017 | 29 parents, 10 grandparents | <6 yrs |  | O/O (13/22) | Country with economic transition, mixed income | Focus group, SSI |
| Ziser 2021 | Germany | NR | 14 mothers, 2 fathers | ~5 yrs |  | O/O (16/16) | Parent with obesity, migrants, low income | SSI |

Study colour in red: ECC-focused. Study colour in blue: O/O-focused.

ECC: early childhood caries; mo: months; NR: not reported; O/O: overweight or obesity; SSI: semi-structured interview; UK: UK; USA: United States of America: yrs: years.
