# Supplementary material for: Carer perspectives on overweight, obesity and dental caries in early childhood: findings from a systematic qualitative review
Source: Front Oral Health. 2025 Jun 18;6:1524715. doi: 10.3389/froh.2025.1524715 (PMC12213562; doi:10.3389/froh.2025.1524715)
Supplement: Supplementary file 6 [file Table6.docx]

Supplementary Files 11 and 12

# Supplementary Table 11. CASP Qualitative Checklist quality appraisal by article (N=98).

| **Reference** | **Clear statement of research aims** | **Appropriate qualitative method** | **Appropriate research design** | **Appropriate recruitment strategy** | **Data collection addressed research issue** | **Researcher-participant relationship** | **Ethical issues** | **Rigorous data analysis** | **Clear statement of findings** | **Results will help locally** |
| --- | --- | --- | --- | --- | --- | --- | --- | --- | --- | --- |
| Amin 2006 | Yes | Yes | Yes | Yes | Unclear | No | Unclear | Yes | Yes | Yes |
| Amin 2009 | Yes | Yes | Yes | Yes | Unclear | Yes | Unclear | Yes | Yes | Yes |
| Amin 2012 | Yes | Yes | Yes | Yes | Unclear | Yes | Unclear | Yes | Yes | Yes |
| Arora 2021 | Yes | Yes | Yes | Yes | Yes | Yes | Yes | Yes | Yes | Yes |
| Athavale 2020 | Yes | Yes | Yes | Unclear | Unclear | No | Yes | No | Unclear | Yes |
| Balasooriyan 2024 | Yes | Yes | Yes | Yes | Yes | Yes | Yes | Yes | Yes | Yes |
| Baughcum 1998 | Yes | Yes | Yes | Yes | Yes | No | Unclear | Yes | Yes | Yes |
| Beck 2018 | Yes | Yes | Yes | Yes | Yes | Unclear | Unclear | Yes | Yes | Yes |
| Bektas 2020 | Yes | Yes | Yes | Yes | Yes | Yes | Yes | Yes | Yes | Yes |
| Bentley 2017 | Yes | Yes | Yes | Yes | Yes | No | Yes | Unclear | Unclear | Yes |
| Brotanek 2009 | Yes | Yes | Yes | Yes | Yes | Unclear | Unclear | Unclear | Yes | Yes |
| Butten 2020 | Yes | Yes | Yes | Yes | Yes | Yes | Yes | Yes | Yes | Yes |
| Cave 2021 | Yes | Yes | Yes | Unclear | No | Unclear | Unclear | No | Yes | Yes |
| Cespedes 2012 | Yes | Yes | Unclear | Yes | Yes | No | Unclear | Yes | Yes | Yes |
| Chaidez 2011 | Yes | Yes | Yes | Yes | Yes | Unclear | Unclear | No | Yes | Yes |
| Cheney 2019 | Yes | Yes | Unclear | Yes | Yes | Unclear | Unclear | Yes | Yes | Yes |
| Collins 2016 | Yes | Yes | Yes | Yes | Yes | Yes | Yes | No | Yes | Yes |
| Crawford 2004 | Yes | Yes | Unclear | Yes | Yes | No | Unclear | No | No | Yes |
| Custodio 2019 | Yes | Yes | Yes | Yes | Yes | No | Yes | Unclear | Yes | Yes |
| Dinkel 2017 | Yes | Yes | Unclear | Yes | Yes | No | Unclear | Yes | Yes | Yes |
| Do 2016 | Yes | Yes | Unclear | Yes | Unclear | No | Yes | Yes | Yes | Yes |
| Ek 2020 | Yes | Yes | Yes | Yes | Yes | Yes | Yes | Yes | Yes | Yes |
| Eli 2014 | Yes | Yes | Yes | Yes | Yes | No | Yes | Yes | Yes | Yes |
| Elwell 2021 | Yes | Yes | Yes | Yes | Yes | Unclear | Unclear | Yes | Yes | Yes |
| Finlayson 2019 (EHS) | Yes | Yes | Yes | Yes | Yes | Unclear | Unclear | Yes | Unclear | Yes |
| Finlayson 2019 (Hispanic) | Yes | Yes | Yes | Yes | Yes | Unclear | Yes | Yes | Yes | Yes |
| Gainsbury 2018 | Yes | Yes | Yes | No | Yes | Unclear | Unclear | Yes | Yes | Yes |
| Galvez 2022 | Yes | Yes | Yes | Yes | Yes | Yes | Yes | Yes | Yes | Yes |
| Glover 2019 | Yes | Yes | Yes | Yes | Unclear | Yes | Yes | Yes | Yes | Yes |
| Goodell 2008 | Yes | Yes | Yes | Yes | Yes | Unclear | Yes | Yes | Yes | Yes |
| Guendelman 2010 | Yes | Yes | Yes | Yes | Yes | No | No | No | Yes | Yes |
| Guerrero 2011 | Yes | Yes | Unclear | Yes | Yes | Unclear | No | Yes | Yes | Yes |
| Hardy 2019 | Yes | Yes | Yes | Yes | Yes | Yes | Yes | Yes | Yes | Yes |
| Hashim 2010 | Yes | Yes | Unclear | Yes | Yes | Unclear | Yes | Yes | Yes | Yes |
| Hilton 2007 | Yes | Yes | Yes | Yes | Yes | Yes | Unclear | Yes | Yes | Yes |
| Hoeft 2010 | Yes | Yes | Yes | Yes | Yes | No | Unclear | Yes | Yes | Yes |
| Hoeft 2015 | Yes | Yes | Yes | Yes | Yes | Unclear | Unclear | Unclear | Unclear | Yes |
| Horowitz 2015 | Yes | Yes | Yes | Yes | Unclear | Unclear | Unclear | Unclear | No | Yes |
| Horowitz 2016 | Yes | Yes | Unclear | Yes | Yes | Unclear | Unclear | No | No | Yes |
| Horton 2008 | Yes | Yes | Yes | Yes | No | Unclear | Unclear | Yes | Yes | Yes |
| Horton 2009 | Yes | Yes | Yes | Yes | Unclear | No | Unclear | Unclear | Yes | Yes |
| Hughes 2010 | Yes | Yes | Yes | Yes | Unclear | No | Unclear | Yes | Yes | Yes |
| Isong 2012 | Yes | Yes | Yes | Yes | Yes | Yes | Yes | Unclear | Yes | Yes |
| Jain 2001 | Yes | Yes | Unclear | Yes | Yes | No | Unclear | Yes | Yes | Yes |
| Karasz 2014 | Yes | Yes | Unclear | Yes | Unclear | No | No | Unclear | Yes | Yes |
| Keenan 2010 | Yes | Yes | Unclear | No | No | No | No | No | No | Unclear |
| Klingberg 2020 | Yes | Yes | Yes | Yes | Yes | Unclear | Yes | Yes | Yes | Yes |
| Lakhanpaul 2020 | Yes | Yes | Yes | Yes | Yes | Yes | Yes | Yes | Yes | Yes |
| Lee 2022 | Yes | Yes | Yes | Yes | Yes | Yes | Yes | Unclear | Yes | Yes |
| Lindsay 2009 | Yes | Yes | Unclear | Yes | Yes | No | Unclear | Yes | Yes | Yes |
| Lindsay 2011 | Yes | Yes | Yes | Yes | Yes | No | Yes | Yes | Yes | Yes |
| Lindsay 2012 | Yes | Yes | Unclear | Yes | Unclear | No | Yes | Yes | Yes | Yes |
| Lindsay 2017 | Yes | Yes | Yes | Yes | Yes | Unclear | Yes | Unclear | Yes | Yes |
| Lopez de Valle 2005 | Yes | Yes | Yes | Unclear | Unclear | No | Yes | No | Unclear | Yes |
| Masterson 2014 | Yes | Yes | Yes | Yes | Unclear | No | Unclear | Unclear | Yes | Yes |
| McDonald 2015 | Yes | Yes | Unclear | Yes | Yes | Yes | Yes | Yes | Yes | Yes |
| McFarren 2020 | Yes | Yes | Yes | Yes | Yes | Yes | Yes | Yes | Yes | Yes |
| McGarvey 2006 | Yes | Yes | Yes | Yes | Yes | Unclear | Yes | Yes | Yes | Yes |
| Mofidi 2009 | Yes | Yes | Yes | Yes | Yes | Unclear | Unclear | Yes | Yes | Yes |
| Momeni 2017 | Yes | Yes | Yes | Yes | Yes | Unclear | Yes | No | Unclear | Yes |
| Momeni 2019 | Yes | Yes | Yes | Yes | Yes | No | Yes | No | No | Yes |
| Naidu 2012 | Yes | Yes | Yes | Yes | Yes | Yes | Yes | Yes | Yes | Yes |
| Nicely 2019 | Yes | Yes | Unclear | Yes | Yes | Yes | Unclear | Yes | Yes | Yes |
| Nicol 2014 | Yes | Yes | Yes | Yes | Yes | Yes | Yes | Yes | Yes | Yes |
| Patino-Fernandez 2013 | Yes | Yes | Yes | Yes | Yes | No | Unclear | Unclear | Yes | Yes |
| Poirer 2021 (barriers) | Yes | Yes | Yes | Yes | Unclear | Yes | Yes | Yes | Yes | Yes |
| Poirer 2021 (motivation) | Yes | Yes | Yes | Yes | Unclear | Yes | Yes | Yes | Yes | Yes |
| Poirier 2022 | Yes | Yes | Yes | Yes | Unclear | Yes | Yes | Yes | Yes | Yes |
| Porter 2016 | Yes | Yes | Unclear | Yes | Yes | No | Unclear | Unclear | No | Yes |
| Prowse 2014 | Yes | Yes | Yes | Yes | Yes | Yes | Unclear | Unclear | Yes | Yes |
| Rachmi 2017 | Yes | Yes | Unclear | Yes | Yes | No | Yes | Yes | Yes | Yes |
| Redsell 2010 | Yes | Yes | Unclear | Yes | Yes | Yes | Yes | Yes | Yes | Yes |
| Rich 2005 | Yes | Yes | Yes | Yes | Yes | No | Unclear | Unclear | Yes | Yes |
| Riggs 2015 | Yes | Yes | Yes | Yes | Yes | Yes | Yes | Unclear | Yes | Yes |
| Rivera 2020 | Yes | Yes | Yes | Yes | Yes | Unclear | Unclear | No | Unclear | Yes |
| Rodríguez-Oliveros 2011 | Yes | Yes | Yes | Yes | Yes | No | Unclear | Unclear | Yes | Yes |
| Roguski 2020 | Yes | Yes | Yes | Yes | Yes | Yes | Unclear | Yes | Yes | Yes |
| Safaiyan 2021 | Yes | Yes | Unclear | Unclear | No | Unclear | Yes | Yes | Unclear | Yes |
| Shrikrishna Suprabha 2022 | Yes | Yes | Yes | Yes | Yes | Yes | Yes | Yes | Yes | Yes |
| Small 2009 | Yes | Yes | Yes | Yes | Unclear | No | Yes | Yes | Yes | Yes |
| Steinman 2010 | Yes | Yes | Yes | Yes | Yes | Yes | Yes | Yes | Yes | Yes |
| Suprabha 2021 | Yes | Yes | Yes | Yes | Yes | Yes | Yes | Yes | Yes | Yes |
| Suprabha 2022 | Yes | Yes | Yes | Yes | Yes | Yes | Yes | Yes | Yes | Yes |
| Suprabha 2024 | Yes | Yes | Yes | Yes | Yes | Yes | Yes | Yes | Yes | Yes |
| Suprawoto 2019 | Yes | Yes | Unclear | Yes | No | No | No | No | Unclear | Unclear |
| Sussner 2008 | Yes | Yes | Yes | Yes | Yes | Yes | Yes | Unclear | Yes | Yes |
| Syrad 2015 | Yes | Yes | Unclear | Yes | Yes | Yes | Yes | Yes | Yes | Yes |
| Thompson 2015 | Yes | Yes | Yes | Yes | Yes | Unclear | No | Unclear | Yes | Yes |
| Tiwari 2017 | Yes | Yes | Unclear | Yes | Yes | Yes | Yes | No | Yes | Yes |
| Tiwari 2021 | Yes | Yes | Unclear | Yes | Unclear | No | Unclear | No | Unclear | Yes |
| Toftemo 2013 | Yes | Yes | Yes | Yes | Unclear | Unclear | Yes | Unclear | Yes | Yes |
| Valencia 2016 | Yes | Yes | Yes | Yes | Unclear | No | Yes | Unclear | Yes | Yes |
| van Nes 2018 | Yes | Yes | Yes | Yes | Yes | No | Yes | Unclear | Yes | Yes |
| Virgo-Milton 2016 | Yes | Yes | Yes | Yes | Yes | No | Yes | Yes | Yes | Yes |
| Weinstein 1999 | Yes | Yes | Yes | Yes | Yes | Yes | No | No | Yes | Yes |
| Woolford 2007 | Yes | Yes | Yes | Yes | Yes | No | Unclear | No | Yes | Yes |
| Wu 2021 | Yes | Yes | Yes | Yes | No | No | Unclear | Yes | Yes | Yes |
| Ziser 2021 | Yes | Yes | Yes | Yes | Yes | Unclear | Yes | No | Yes | Yes |

# Supplementary Figure 1. Summary of CASP Qualitative Checklist quality appraisal (N=98).

**
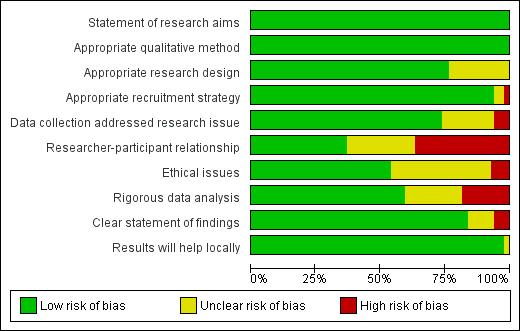
**
