# Supplementary material for: Carer perspectives on overweight, obesity and dental caries in early childhood: findings from a systematic qualitative review
Source: Front Oral Health. 2025 Jun 18;6:1524715. doi: 10.3389/froh.2025.1524715 (PMC12213562; doi:10.3389/froh.2025.1524715)
Supplement: Supplementary file 7 [file Table7.docx]

Supplementary File 13

# Supplementary Table 12. Representative quotes of thematic findings.

| **Content addressed** | **Quote, O/O research** | **Quote, ECC research** |
| --- | --- | --- |
| **Theme 1: How parents view a healthy child** | | |
| Theme 1 | *… nice and open and they’re not scared… and they speak their mind.* (Grandmother with 6 month-5 year old grandchild, population at increased risk of childhood O/O)^1^ | *A healthy baby? Smiling, walking, eating and sleeping well, laughing… happy.* (Mother with 6-24 month old child, population at increased risk of ECC)^2^ |
| Theme 1.1 (health of primary teeth) | N/A | *I know [primary] teeth are very important. If they get ‘rot’ they would destroy the second set; it’s also related to their digestion and nutrition.* (Parent with ≤6 year old child with ECC)^3^  *[Primary] teeth are important, but […] better to have cavities in your [primary] teeth, they will shed anyway, than in the permanent teeth.* (Mother with ≤5 year old child, population at increased risk of ECC)^4^ |
| Theme 1.1 (healthy weight based on body size) | *Oh, we always talk about how big they are and they are always showing their muscles and stuff like that. We encourage them to eat their veggies so then they can get big muscles and then they want to show off their muscles.* (Father with 3-5 year old child with overweight)^5^  *I think that she's very thin, but the doctor told me that she's okay… that everything's okay, normal for height and weight… I'm relaxed about it because if [the doctor] tells me that it's because they know, so I have calmed down.* (Parent with 4-24 month old child, population at increased risk of childhood O/O)^6^ | N/A |
| Theme 1.2.2 | *We had one girl in the [Maori preschool] I was working in, and because her parents were overweight, obese. […] They put their child on a diet and that was torture for us to see the rest of the kids eating yummy [food], and she’s got like carrot sticks and, you know, all this other healthy stuﬀ… So, we’d just like put her lunch away and give her a cake! [laughing] Because that is torture, it really is. You’re punishing the child because of your actions, you know?* (Caregiver of children aged 6 months-4 years, population at increased risk of childhood O/O)^1^ | *The doctors in this country forbade us to force feed the babies… But [as his mother] I know that it has been three hours and he must be hungry, so I feed him again.* (Mother with 6-24 month old child, population at increased risk of ECC)^2^ |
| **Theme 2: How parents understood unhealthy teeth and weight** | | |
| Theme 2.1 | N/A | *I thought that he was just making it up because I would check his teeth and I would not be able to see the cavities... I would tell him, ‘How can your molar be hurting?’ Until one day he had this big bump—it’s a blister filled with pus and that side of his face was swollen, and I took him in to the dentist, and that’s when they told me that he had a bad infection.* (Mother with 5 year old child with ECC)^7^ |
| Theme 2.2 | *Normal kids my son’s age are wearing a size that they’re supposed to be wearing. My [3 year old] son is wearing sizes that a seven- or eight-year-old child would wear.* (Mother with 3 year old child with overweight)^8^  *That’s one thing I don’t think they should go by* [to identify overweight or obesity]*, is their growth charts, because they’re just doing it, basing it all upon what the average child is supposed to be. Nobody knows exactly what the average child is supposed to be.* (Mother with ≤5 year old child with overweight)^8^ | N/A |
| Theme 2.3 | *If he sees that I am going to drink soda, he knows that he drinks juice and wants juice. If he sees that I keep juice where he can reach it… he goes and opens the door and asks that I give him juice. When we go to McDonald’s, I pour out the soda and give him juice. His juice in the cup as if we were all drinking the same thing.* (Mother with 12-46 month old child, population at increased risk of childhood O/O)^9^ | *He was eating a lot of candy, but I took him off that. I only give him ice cream bars now. I don’t want him to eat candy, but when I took him back [to the dentist] he had more caries. I don’t know why. Who knows why? I was asking myself, ‘How he could have more?’.* (Parent with ≤5 year old child with ECC)^10^  *All types of foods are good, and all have nutritional values. Sugar should not be an exception, because it provides a lot of energy to the children.* (Mother with 5 year old child, population at increased risk of ECC)^11^ |
| Theme 2.4 (consequences of ECC or O/O) | *At the [Women, Infants, and Children’s food and nutrition program]… they say that she’s overweight. But I see her being very active. She likes to run, she does a lot of exercise, and I don’t see her very fat. […] This is where I have doubt… that she is overweight.* (Mother with 2-5 year old child with O/O)^12^ | *[…] if she’s got tooth decay now [in her primary teeth], at least she’s got a second set coming but the set you have for life, that’s it.* (Mother with 2-3 year old child, population at increased risk of ECC)^13^  *Only when [my daughter] was told by the dentist that the [permanent] teeth may not form properly and that she wouldn’t be as beautiful, did I think of brushing and care of the [primary] teeth as important and when the dentist explained this teeth should be kept until the [permanent] tooth comes out, otherwise she has a very ugly teeth […].* (Mother with ≤6 year old child with ECC)^14^ |
| Theme 2.4 (significance of ECC or O/O) | *[while I breast fed] I felt that my son was healthy… but then my mother-in-law came and gave him juice, and baby food… since my mother-in-law is a little heavy, they told me ‘chubby children look cuter’, and then my son got to the point where he couldn’t walk… he could walk, but my son was really fat.* (Mother with 2-5 year old child with O/O)^15^ | *When he wants to eat, he can’t eat hard things […] he cannot chew well and I think that affects his daily life as a child […] he needs his good little teeth […] now he complains that a molar hurts him […] he doesn’t want to eat, I had to take him to the doctor again.* (Mother with 2-5 year old child with ECC)^16^ |
| **Theme 3: Supporting parents to maintain their child’s health** | | |
| Theme 3.1 (attitude to information sources) | *In [Women’s, Infant’s and Children’s food and nutrition program]… they tell you not to give solid foods until six, seven months. I say at that age they are very big just to be starting to eat. I never paid attention to that. I have seen many children who don’t know how to eat and when they give them food, they get sick from stomach problems.* (Mother with 12-46 month old child, population at increased risk of childhood O/O)^9^ | *[Early Head Start early childhood education staff] can give us advice, but this is our child so we’re going to still do it the way that we believe is right. […] I’ll listen to the advice that they give me, but I’m still going to have my own opinions about it, and deal with that in my manner.* (Parent with ≤3 year old child, population at increased risk of ECC)^17^ |
| Theme 3.1 (attitude from information sources) | *Her doctor has always said that she’s very healthy; she’s really bright and wants to learn everything and she’s still very physically active. […] And so that has encouraged me that her weight is okay and her doctor has always said that she’s just fine.* (Mother with 2-5 year old child with obesity)^5^ | *[The] family doctor, when my child’s tooth was a little black, he told me to go to dentist—but no other information. The dentist said there’s nothing too bad about the teeth—it’s just the colour. And when her new teeth come out, they’ll be better. He said to brush all the time and I do not have to feed her by the bottle.* (Mother with <6 year old child with ECC)^18^ |
| Theme 3.1 (conflicting information) | *But at the Head Start [early childhood education program], whenever they do his weight, his height and all that, they sent me a letter that he's overweight. When I take him to the [Women’s, Infant’s and Children’s food and nutrition program] office, it's also that he's overweight. You kind of have to trust the doctor [who stated her son’s weight was not an issue] more… you kind of have more confidence in the doctor.* (Mother with 4 year old child with O/O)^19^ | *When my kids start to have tooth decay, I usually ask for advice from my relatives. They always say that bad teeth would fall off by themselves and extraction would not be needed. I really cannot make up my mind as to whether I should take my kids to a dentist or not.* (Mother with 1-5 year old child with ECC, population at increased risk of ECC)^20^ |
| Theme 3.2 | *I asked the doctor if he’d send me to the nutritionist for an [health education session], and he told me no, that I should just smiddle feeding him foods that I consider bad… I think there’s a lack of information… we’re not feeling well-oriented [to nutrition].* (Parent with 2-5 year old child with O/O)^15^ | *You know, they give us juice on [Women’s, Infant’s and Children’s food and nutrition program]. They don't give us… we get the voucher that we can use however we want for fresh fruits and vegetables, but 8 bucks [for fresh food vouchers]…* (Parent with 6-36 month old child, population at increased risk of ECC)^21^ |
| Theme 3.3 (parents’ ability to support environment for healthy weight or teeth) | *But I think if we was to change his diet, maybe that may have a lot to do with it, but he’s at a picky stage to where I couldn’t change his diet if I wanted to because it’s what he wants to eat or he don’t eat nothing at all.* (Mother of 2.5-5 year old child with O/O)^8^  *Well, now he asks [for ice cream] […] where I live, the ice cream vendors with the carts pass by. And every time they pass by, he is outside and hears it and knows. And then he says ‘ice cream, ice cream,’ so I have to buy it for him.* (Mother of 4-24 month old child, population at increased risk of O/O)^6^ | *[…] with my daughter, in the beginning when we really first started brushing it was really hard. I used to get really frustrated, and sometimes... There'd be days where I'm like, ‘You know what? Time to go to bed.’* (Parent with 6-36 month old child, population at increased risk of ECC)^21^  *The nurse told her that toddlers should start cleaning their teeth when they are two years old, she doesn’t believe in that and she hasn’t applied it to her children… the child is still too young to look after his or herself and she believes to wait until the child is five years old, when the child can be independent to clean their teeth.* (Interpreter for mother of young child, population at increased risk of ECC)^22^ |
| Theme 3.3 (being undermined by other carers) | *He drinks a lot of bottled juices and eats French fries… a lot of sweets. When he’s with his grandmother, she is very permissive […] buys him everything he asks for […] promotes his bad habits. She tells me, ‘My role is to say ‘yes’ to him, your role is to educate him; so don’t say anything to me when I am with him’.* (Parent with 2-5 year old child with O/O)^15^ | *We get a lot of the, ‘oh he’ll be fine—they’re just his baby teeth, they’re not his adult teeth, he gets to start over in a few years’ [from the child’s grandmother]. Because she doesn’t like to see us tell him he can’t have something.* (Mother of ≤6 year old child with ECC)^16^ |
| Theme 3.3 (parents’ response to child hunger maintains obesogenic or cariogenic behaviour) | *You can tell. Every time you give them a bottle… and then they get to crying again a half hour later, then you give them another bottle and they take that, they are not gettin’ full enough. That’s how I think.* (Mother with 1-3 year old child, population at increased risk of childhood O/O) ^23^  *He’s not happy unless he has a bottle in his mouth… I don’t want to starve him, but you know, I don’t know what I can do.* (Mother with 2-5 year old child with overweight)^24^ | *If I feed my child table food, I don’t know exactly how much he has eaten. If she is drinking from a glass then she will spill it – and how can I tell how much she has had in total? If she feeds from the bottle then the amount consumed can be seen. […] My child doesn’t want to eat rice, and then the quantity of milk consumption goes up. I give her the [bottle] thrice a day if she doesn’t want to eat rice. Around 2-3 am in the night, she needs to be fed with the [bottle].* (Mother with 6-24 month old child, population at increased risk of ECC)^2^ |

ECC: early childhood caries; N/A: not applicable; O/O: overweight/obesity

**References**

1. Glover M, Wong SF, Taylor RW, et al. The Complexity of Food Provisioning Decisions by Māori Caregivers to Ensure the Happiness and Health of Their Children. *Nutrients.* 2019; 11(5):994-994. doi:10.3390/nu11050994.

2. Karasz A, Patel V, Ranasinghe S, Chaudhuri K, McKee D. Preventing caries in young children of immigrant Bangladeshi families in New York: Perspectives of mothers and paediatricians. *Community Dent Health.* 2014; 31(2):80-84.

3. Amin MS, Harrison RL, Weinstein P. A qualitative look at parents' experience of their child's dental general anaesthesia. *Int J Paediatr Dent.* 2006; 16(5):309-319. doi:10.1111/j.1365-263X.2006.00750.x.

4. van Nes KA, Veerkamp JSJ, Reis R. Barriers and opportunities to oral health in Dutch-Moroccan children in the Netherlands: a narrative report. *Eur Arch Paediatr Dent.* 2018; 19(5):353-364. doi:10.1007/s40368-018-0367-3.

5. Eli K, Howell K, Fisher PA, Nowicka P. “A little on the heavy side”: a qualitative analysis of parents' and grandparents' perceptions of preschoolers' body weights. *BMJ Open.* 2014; 4(12):e006609. doi:10.1136/bmjopen-2014-006609.

6. Beck AL, Hoeft KS, Takayama JI, Barker JC. Beliefs and practices regarding solid food introduction among Latino parents in Northern California. *Appetite.* 2018; 120:381-387. doi:10.1016/j.appet.2017.09.023.

7. Horton S, Barker JC. Rural Mexican immigrant parents' interpretation of children's dental symptoms and decisions to seek treatment. *Community Dent Health.* 2009; 26(4):216-221. doi:10.1922/CDH 2320Horton06.

8. Hughes CC, Sherman SN, Whitaker RC. How low-income mothers with overweight preschool children make sense of obesity. *Qual Health Res.* 2010; 20(4):465-478. doi:10.1177/1049732310361246.

9. Chaidez V, Townsend M, Kaiser LL. Toddler-feeding practices among Mexican American mothers. A qualitative study. *Appetite.* 2011; 56(3):629-632. doi:10.1016/j.appet.2011.02.015.

10. Hoeft KS, Barker JC, Masterson EE. Urban Mexican-American mothers' beliefs about caries etiology in children. *Community Dent Oral Epidemiol.* 2010; 38(3):244-255. doi:10.1111/j.1600-0528.2009.00528.x.

11. Hashim R, Fitzgerald RP, Schafer CT, Thomson WM. Mothers' understanding of dental-caries related feeding practices and children's use of dental care in Ajman. *Social Science and Dentistry.* 2010; 1(2):97-107.

12. Guerrero A, Slusser W, Barreto P, Rosales N, Kuo A. Latina Mothers' Perceptions of Healthcare Professional Weight Assessments of Preschool-Aged Children. *Matern Child Health J.* 2011; 15(8):1308-1315. doi:10.1007/s10995-010-0683-7.

13. Arora A, Lucas D, To M, et al. How Do Mothers Living in Socially Deprived Communities Perceive Oral Health of Young Children? A Qualitative Study. *Int J Environ Res Public Health.* 2021; 18(7):3521. doi:10.3390/ijerph18073521.

14. Nicol P, Al-Hanbali A, King N, Slack-Smith L, Cherian S. Informing a culturally appropriate approach to oral health and dental care for pre-school refugee children: a community participatory study. *BMC Oral Health.* 2014; 14:69. doi:10.1186/1472-6831-14-69.

15. Cespedes E, Andrade GOM, Rodríguez-Oliveros G, et al. Opportunities to Strengthen Childhood Obesity Prevention in Two Mexican Health Care Settings. *Int J Paediatr Dent.* 2012; 2(3):496-504.

16. Isong IA, Luff D, Perrin JM, Winickoff JP, Ng MW. Parental Perspectives of Early Childhood Caries. *Clin Pediatr (Phila).* 2012; 51(1):77-85. doi:10.1177/0009922811417856.

17. Mofidi M, Zeldin LP, Rozier RG. Oral health of Early Head Start children: a qualitative study of staff, parents, and pregnant women. *Am J Public Health.* 2009; 99(2):245-251. doi:10.2105/AJPH.2008.133827.

18. Prowse S, Schroth RJ, Wilson A, et al. Diversity Considerations for Promoting Early Childhood Oral Health: A Pilot Study. *Int J Dent.* 2014; 2014:175084. doi:10.1155/2014/175084.

19. McDonald S. The Lived Experiences of Mexican-Heritage Mothers Caring for Overweight Preschool Children. *Qual Rep.* 2015; 20(4):431-450. doi:10.46743/2160-3715/2015.2119.

20. Hilton IV, Stephen S, Barker JC, Weintraub JA. Cultural factors and children's oral health care: a qualitative study of carers of young children. *Community Dent Oral Epidemiol.* 2007; 35(6):429-438. doi:10.1111/j.1600-0528.2006.00356.x.

21. Collins CC, Villa-Torres L, Sams LD, Zeldin LP, Divaris K. Framing Young Childrens Oral Health: A Participatory Action Research Project. *PLoS One.* 2016; 11(8):e0161728. doi:10.1371/journal.pone.0161728.

22. Riggs E, Gibbs L, Kilpatrick N, et al. Breaking down the barriers: a qualitative study to understand child oral health in refugee and migrant communities in Australia. *Ethn Health.* 2015; 20(3):241-257. doi:10.1080/13557858.2014.907391.

23. Baughcum AE, Burklow KA, Deeks CM, Powers SW, Whitaker RC. Maternal feeding practices and childhood obesity: a focus group study of low-income mothers. *Arch Pediatr Adolesc Med.* 1998; 152(10):1010-1014. doi:10.1001/archpedi.152.10.1010.

24. Jain A, Sherman SN, Chamberlin LA, Carter Y, Powers SW, Whitaker RC. Why don't low-income mothers worry about their preschoolers being overweight? *Pediatrics.* 2001; 107(5):1138-1146. doi:10.1542/peds.107.5.1138.
